# Supplementary material for: Intrinsic timescale develops hierarchically from sensorimotor to association cortex in youth and stabilizes in adulthood
Source: PLoS Biol. 2026 Sep 29;24(9):e3004037. doi: 10.1371/journal.pbio.3004037 (PMC13638041; doi:10.1371/journal.pbio.3004037)
Supplement: Supplementary file 1 [file pbio.3004037.s014.docx]

## Editor:

*As you will see below, the reviewers write that study is overall well executed and provides potentially important insights. However, they also raise a number of concerns that need to be addressed. For Reviewer 1 and 2, the most important comments are on the discrepancies with prior work on intrinsic timescale development. There are also concerns about the use of a different metric by Reviewer 3, and about the framing/terminology around “development changes” in this cross-sectional study.*

Dear Dr. Schnell and Reviewers,

Thank you for the constructive feedback on our initial submission and for the opportunity to revise and resubmit the manuscript. Following the Reviewers’ comments and suggestions, we have thoroughly revised the manuscript. Here we respond to each of the Reviewers’ comments in detail. Reviewer comments are in *italics* and our responses are in regular font. New text is indicated by blue font.

Highlights include:

(1) We repeated the main analyses using the lag-1 temporal autocorrelation (lag-1 TA) metric employed by Bero et al. (2026), allowing a direct comparison with prior work and providing an additional intrinsic timescale metric alongside the measures included in our original submission.
(2) We decomposed the sum of positive autocorrelation into short- and long-lag contributions, showing that lag-1 TA primarily reflects short-lag autocorrelation, whereas our original intrinsic timescale measure is more sensitive to longer-lag positive autocorrelation.
(3) We revised the manuscript to emphasize the cross-sectional nature of the study, modifying the terminology throughout to avoid implying longitudinal developmental changes. Specifically, we have changed all instances of “developmental changes” and “age-related changes” in the original manuscript to “developmental patterns” and “age-related differences” in the revised manuscript. These changes are indicated by blue font in the revised manuscript. Furthermore, in addition to mentioning the cross-sectional nature of our study as a limitation, we have now also indicated this information at multiple parts of the revised manuscript:

Abstract:

Here we estimate the intrinsic timescale in two independent cross-sectional datasets of youth (HCPD: *n*=565; HBN: *n*=729; age range 8–22 years) and investigate its neurodevelopmental patterns.

“Results” section, paragraph #1:

Cortical maps of intrinsic timescale were estimated using the autocorrelation function of resting-state fMRI time series for two cross-sectional, developmental cohorts (ages 8–22 years) and a young-adult dataset (ages 22–37 years).

“Methods” section, “Participants” subsection:

We used resting-state functional magnetic resonance imaging (fMRI) data from 3 independent cohorts: (1) a cross-sectional developmental dataset used as a discovery sample; (2) an independent cross-sectional developmental dataset used to replicate the findings; (3) a young adult dataset to evaluate the specificity of our results.

## Reviewer #1 (Jesse T. Miles):

Summary of the research and overall impression

*This manuscript reports a developmental shift in intrinsic neural timescales organized around the sensorimotor-to-association neural hierarchy, otherwise known as the SA axis. Specifically, the authors report an increase in neural timescales throughout childhood and adolescence which demonstrates stronger age-dependence in association cortex, effect-size increases that recapitulate the SA axis, and greater timescale stability in sensorimotor regions. Based on these results the authors claim that sensorimotor region intrinsic neural timescales are relatively stable throughout childhood and adolescence, while timescales increase in association regions.*

*A strength of the manuscript is that the authors reproduce their findings using two large, independent developmental fMRI datasets, and show that the age-dependence does not continue into young adulthood in a third dataset. The authors also diligently varied analytic workflows, and in doing so demonstrated that their conclusions were robust to a variety of possible demographic and analytic confounds. In addition, the manuscript is well-organized, well-researched, and impressively up-to-date.*

*However, more discussion of the fact that the conclusions seem to differ from prior work on intrinsic timescale development is warranted. The authors point out certain methodological and demographic differences between their work and others, but the studies cited used a wide variety of neural recording modalities, preprocessing techniques, analytic pipelines, and age ranges to come to broadly similar conclusions (i.e., timescales generally decrease with age, but may do so heterogeneously across the cortex in ways that align with SA axis maturation). Because of the broad interest of the work, its efforts toward methodological rigor, and its potential novelty, I recommend the manuscript be accepted. However, explaining and recontextualizing its findings may require major revision.*

We thank the Reviewer for their kind words and helpful comments! We have now substantially revised the manuscript in response to their comments, as detailed below.

---

*Specific areas for improvement*

*1. The Bero et al., 2026 study that the authors cite did not find relationships between childhood timescales and the S-A axis (figure 13), despite finding that some frontal regions may increase their temporal timescales throughout childhood (figure 8). While many frontal regions rank high on the SA axis, other high-ranking regions are not in the frontal cortex. Plus, Bero et al. report generally faster timescales in adults than children (figure 2), even in many networks that contain frontal regions. The authors point out that Bero et al. used a different timescale measure, which could account for some differences, but the analytic methods seem similar enough that I'm surprised the conclusions differ. Can the authors comment on whether they, too, see their results as primarily applicable to frontal regions instead of association regions, broadly? Aside from methodological differences, are there other reasons that could explain the differences between their findings and Bero et al.'s?*

*>Suggested analyses to strengthen the link to Bero et al., 2026:*

*Running a supplemental analysis using the lag-1 autocorrelation. The authors already compare their method to fitting an exponential decay and to calculating the first zero-crossing of the ACF window, so they seem to already be set up to make the lag-1 comparison. I also think it's important to run at least one dataset through the entire analysis, not just compare the timescales output by the different methods. Essentially, reproduce figure 3 with the lag-1 timescales to establish whether Bero et al.'s method would lead to a different conclusion. If it doesn't then clearly there is more driving the differences than the timescale calculation.*

Thank you for this thoughtful comment and extremely helpful suggestion. We agree that the comparison with Bero et al. (2026) is important. In our original analysis using the ACF-sum timescale, we interpret the developmental patterns as broadly organized along the sensorimotor-association (S-A) axis. Specifically, age-related increases were strongest in high-ranking association regions along the S-A axis, while sensorimotor regions remained relatively stable. However, we agree with the Reviewer that Bero et al. report a somewhat different pattern, with decreases in sensorimotor cortex and increases most prominently in prefrontal regions.

Following the Reviewer’s suggestion, we therefore performed a new supplementary analysis in HCPD using lag-1 temporal autocorrelation (lag-1 TA), following the same approach as Bero et al. (2026). We ran this analysis through the full pipeline, rather than only comparing the final timescale maps, as suggested by the Reviewer. Similar to our original findings, we found a trend-like positive association between whole-brain average lag-1 TA and participant age (**Figure S9a**). At the regional level, lag-1 TA showed both age-related increases and decreases across cortex (**Figure S9b**), with age-related increases most prominently visible in frontal/prefrontal regions, partially consistent with Bero et al. However, the regional age effects estimated using lag-1 TA did not follow the S-A axis as in our original ACF-sum analysis (**Figure S9c**; *r*_s_ = -0.005, *p*_spin_ = 0.9).

We also directly compared lag-1 TA with our original ACF-sum timescale metric. The mean regional timescale maps estimated using the two approaches were not significantly correlated (**Figure S10a**; *r*_s_ = 0.17, *p*_spin_ = 0.23), whereas the regional age effects estimated from the two measures were moderately correlated (**Figure S10b**; *r*_s_ = 0.55, *p*_spin_ = 0.0001). These findings suggest that within the same HCPD sample, preprocessing pipeline, parcellation, and statistical framework, lag-1 TA and ACF-sum timescale differ in their mean spatial distribution but show partially overlapping developmental effects.

Given these analyses, we do not view our original findings as applying only to the frontal cortex. Rather, using the original ACF-sum measure, the strongest age-related increases were distributed across higher-ranking association regions along the S-A axis. Additionally, using Bero et al.’s metric in our HCPD sample, we observed age-related increases that were most apparent in frontal/prefrontal regions, although these increases were not solely restricted to the prefrontal cortex. We therefore agree that the choice of timescale metric may contribute to the differences across studies. However, our new analysis also suggests that it is unlikely to be the only source of discrepancy.

We speculate that the remaining discrepancies may partly reflect several methodological differences between the studies. First, Bero et al. included participants aged 5-22 years and used a mean FD threshold of 0.34 mm, whereas our sample included participants aged 8-22 years and used a stricter mean FD threshold of 0.2 mm. Second, Bero et al. used minimally processed HCPD data with ICA-FIX denoising and high-pass filtering, followed by an additional high-pass filter at 0.008 Hz applied to the parcellated time series. In contrast, we processed the raw HCPD data using fMRIPrep and XCP-D, including band-pass filtering at 0.01-0.08 Hz. Because lag-1 TA is sensitive to short-lag smoothness, differences in temporal filtering may influence the amount of high-frequency variance retained in the BOLD signal and therefore affect the metric and its age-related patterns. Third, Bero et al. used linear regression models to estimate age effects, whereas we used generalized additive models (GAMs) to assess both linear and nonlinear developmental effects.

In addition, following a helpful suggestion from Reviewer 2, we performed a complementary analysis to better understand why lag-1 TA and ACF-sum timescale may diverge even when applied to the same fMRI data. Specifically, we decomposed the ACF-sum timescale into short- and long-lag components by first identifying a data-driven elbow point in the group-average ACF, and then summing the positive ACF values separately before and after this elbow (detailed below, and in the Methods section of the manuscript). This analysis showed that lag-1 TA was most strongly associated with the short-lag component of the ACF, whereas our original ACF-sum timescale was more strongly associated with the long-lag component (**Figure S11**). Thus, different timescale metrics may emphasize different characteristics of the same fMRI signal. We speculate that lag-1 TA primarily indexes short-lag temporal persistence, whereas ACF-sum timescale is more sensitive to sustained positive autocorrelation across longer lags. More broadly, this divergence may reflect developmental differences in the shape of the ACF itself: lag-1 TA captures only the immediate rate of decorrelation (i.e., only at lag 1), whereas the ACF-sum measure is sensitive to broader features of the ACF curve, including longer positive tails across later lags.

We have now made several modifications to the manuscript to reflect on these points:

“Methods” section:

**Intrinsic timescale estimated using lag-1 temporal autocorrelation**

As a complementary estimate of intrinsic timescale, we calculated lag-1 temporal autocorrelation (lag-1 TA) for each brain region and participant, following the approach used by Shinn et al. (2023) and Bero et al. (2026). Lag-1 TA quantifies the similarity between a time series and a copy of itself shifted by one time point. In the context of fMRI, this corresponds to the correlation between the BOLD signal at time point ***t*** and the BOLD signal at the immediately following time point, ***t***+1 , within the same brain region. Higher lag-1 TA values therefore indicate slower decorrelation, or greater temporal persistence, across consecutive time points, whereas lower values indicate faster decorrelation from one time point to the next.

Fully processed parcellated fMRI time series were first z-score normalized within each parcel. We then estimated lag-1 TA using the temporal_autocorrelation(x) function from the “spatiotemporal” Python package (Shinn et al., 2023). This function computes TA-𝚫_1_ , defined as the first-order temporal autocorrelation between consecutive time points. For each participant, the input was a parcel-by-time matrix, and the output was a vector of lag-1 TA values across parcels. Unlike the ACF-sum timescale measure, lag-1 TA captures *only* the first nonzero lag of the ACF rather than the area under the positive portion of the ACF. Accordingly, lag-1 TA was not integrated across multiple lags and was not multiplied by TR; it is a dimensionless autocorrelation coefficient. We repeated subsequent analyses using these parcellated lag-1 TA maps to compare regional developmental patterns with those estimated using the original ACF-sum timescale metric.

**Short- and long-lag contributions to intrinsic timescale**

To examine whether the ACF-sum measure of intrinsic timescale was differentially driven by short- versus longer-lag autocorrelation structure, we decomposed the positive ACF area into short- and long-lag components. First, using the normalized parcellated fMRI time series, we estimated the ACF for each parcel and participant as described above. We then computed a group-average ACF by averaging ACF curves across all parcels and participants. To identify a data-driven lag separating the steep early decay of the ACF from the slower positive tail, we fit a two-piece linear model to the positive portion of the group-average ACF. Specifically, for each candidate lag ***k***, we fit one linear function to the early-lag portion of the group-average ACF and a second linear function to the later-lag portion. We selected the lag ***k*** that minimized the total sum of squared errors across the two fitted line segments. Lag 0 was excluded from this elbow estimation because the ACF at lag 0 is fixed at 1. We searched candidate elbow lags from ***k*** = 3 to 47 within the positive group-average ACF, corresponding to 2.4-37.6 s in HCPD (TR = 0.8 s). The two-piece linear fit identified an elbow at lag ***k*** = 5, corresponding to 4.0 s in HCPD.

Using this single group-level elbow lag ***k***, we then returned to the participant- and parcel-level ACFs and calculated two complementary timescale estimates. The short-lag component, Int_short_ , was calculated as the sum of ACF values from lag 0 through lag ***k***, bounded by the first zero-crossing point when the ACF crossed zero before lag ***k***. The long-lag component, Int_long_ , was calculated as the sum of positive ACF values after lag ***k*** and up to the first zero-crossing point. Both components were multiplied by the dataset-specific TR to express them in seconds. This decomposition ensured that Int_short_ and Int_long_ summed to yield the original positive ACF-sum intrinsic timescale estimate for each parcel and participant. We used this approach to test whether short- and long-lag ACF components captured distinct aspects of development, and specifically whether lag-1 autocorrelation preferentially reflected the short-lag component of the ACF.

“Results” section, “Sensitivity analysis” subsection, paragraph #2:

To test whether intrinsic timescale estimates were sensitive to methodological choices and to better contextualize our findings with prior work, we compared our ACF-sum measure with several alternative approaches for quantifying intrinsic timescale in HCPD. First, we quantified intrinsic timescale using two other approaches based on full ACF: (1) fitting an exponential function to ACF and using the exponential decay constant to estimate intrinsic timescale (Ito et al., 2020); (2) directly using the first zero-crossing point of ACF as intrinsic timescale. Both approaches generated intrinsic timescale maps and developmental patterns consistent with the original method (**Figure S8**). We further repeated the HCPD analyses using lag-1 temporal autocorrelation (lag-1 TA), following prior work (Shinn et al., 2023; Bero et al., 2026). We found that lag-1 TA and ACF-sum timescale differed in their mean spatial distribution but showed partially overlapping developmental effects (**Figures S9-S10**). We also decomposed the ACF-sum timescale into short- and long-lag components and found that lag-1 TA was most closely related to short-lag autocorrelation, whereas the full ACF-sum measure was more strongly influenced by longer-lag positive autocorrelation (**Figure S11**). Together, these analyses suggest that our core findings are robust across several full-ACF-based timescale definitions, while also showing that different metrics emphasize different features of temporal persistence: lag-1 TA primarily reflects short-lag autocorrelation, whereas the ACF-sum measure is more sensitive to longer-lag positive autocorrelation across the full ACF. This suggests that the developmental effects observed with our ACF-sum measure were driven primarily by long-lag ACF structure.

“Discussion” section, new paragraph #4:

To further investigate these differences, we repeated our developmental analyses using lag-1 TA in the same HCPD sample and preprocessing pipeline used for our primary ACF-sum analyses. This comparison showed that lag-1 TA and ACF-sum timescale had distinct regional mean maps but demonstrated partially overlapping regional age effects. Decomposing the ACF-sum timescale into short- and long-lag components further suggested that lag-1 TA and ACF-sum timescale emphasize partially distinct features of the ACF: lag-1 TA primarily reflects immediate short-lag decorrelation, whereas ACF-sum timescale is more sensitive to the broader shape of the ACF, including sustained positive autocorrelation across longer lags. Thus, while methodological differences in preprocessing, sample selection, and modeling may contribute to discrepancies across studies, our results indicate that some differences may also arise because commonly used timescale metrics emphasize different characteristics of the same fMRI signal.

**Figure S9. Developmental patterns of lag-1 temporal autocorrelation in HCPD |** Lag-1 temporal autocorrelation (lag-1 TA) was estimated in HCPD as a complementary measure of intrinsic timescale. **(a)** Cortical map of lag-1 TA and relationship between whole-brain average lag-1 TA and age. Each data point in the scatter plot represents an individual participant (partial *R*^2^ = 0.008, *p*_anova_ = 0.03). **(b)** Region-wise GAMs identified heterogeneous age effects (i.e., partial *R*^2^) across cortex. FDR-corrected maps show regions with significant age effects (*p*_FDR_ < 0.05; 28.2% of regions with significant age effects). **(c)** Age effects on lag-1 TA were not significantly associated with the S-A axis. Significance of the association between age effects and S-A axis rank was assessed using 10,000 spin tests. *r*_s_ denotes Spearman’s rank correlation coefficient. Linear regression line is added for visualization purposes only. **(d)** Regional trajectories of developmental patterns of lag-1 TA were obtained from region-wise GAM results. Each line corresponds to the model fit of each cortical region and is colored based on the region’s rank along the S-A axis.

**Figure S10. Relationship between ACF-sum intrinsic timescale and lag-1 temporal autocorrelation |** We compared regional maps of intrinsic timescale estimated using the original ACF-sum method and lag-1 temporal autocorrelation (lag-1 TA) in HCPD. **(a)** Regional intrinsic timescale maps estimated using the two approaches were not significantly correlated. **(b)** In contrast, regional age effects (i.e., partial *R*^2^) estimated using the two approaches were significantly correlated. Each data point in the scatter plot represents a brain region from the Schaefer 400 atlas. The data points are colored based on their rankings on the S-A axis. *r*_s_ denotes Spearman’s rank correlation coefficient. Significance was assessed using 10,000 spin tests. Linear regression lines are added for visualization purposes only.

**Figure S11. Short- and long-lag contributions to ACF-sum intrinsic timescale |** To examine whether ACF-sum intrinsic timescale was differentially driven by short- versus longer-lag autocorrelation structure, we decomposed the positive ACF area into short-lag and long-lag components. **(a)** The short-lag component was modestly associated with the intrinsic timescale estimated using the original ACF-sum method. The regional age effects from the short-lag component were moderately associated with age effects from the original method. **(b)** In contrast, the short-lag component was highly associated with lag-1 temporal autocorrelation (lag-1 TA), both for regional timescale maps and regional age effects. **(c)** The long-lag component and its age effects were strongly associated with the original findings estimated using the ACF-sum method. **(d)** In contrast, the long-lag component was not significantly associated with lag-1 TA in regional timescale maps and was only weakly associated with lag-1 TA regional age effects. Each data point in the scatter plot represents a brain region from the Schaefer 400 atlas. The data points are colored based on their rankings on the S-A axis. *r*_s_ denotes Spearman’s rank correlation coefficient. Significance was assessed using 10,000 spin tests. Linear regression lines are added for visualization purposes only.

*2. The authors mention that Truzzi and Cusack, 2023, found differences in the timescale hierarchy between infants and adults, and found that adult timescales were generally much shorter. In this manuscript, the authors report similar timescale hierarchies between adults and children/adolescents, while Truzzi and Cusack 2023 suggest that timescales decrease globally. The authors seem to suggest that this is because the SA axis becomes more developed between the neonatal period and middle childhood, giving it "adult-like hierarchical ordering". Similar results to Truzzi and Cusack, 2023 were found in McKeon et al., 2025, who used a similar age range in their EEG and sEEG studies, and Miles et al., 2026, who found association region timescale decreases throughout adolescence. Why, then, do the authors think that they see timescale increases in association regions?*

We agree with the Reviewer that our findings appear to differ from studies reporting decreases in intrinsic timescale during development. We have now expanded the Discussion to acknowledge this discrepancy more explicitly and to offer a possible, though necessarily speculative, interpretation. One possibility is that these differences reflect not only methodological choices, but also the fact that different modalities and timescale metrics may capture different temporal properties of the signal. Specifically, our ACF-sum measure was applied to band-pass filtered (0.01-0.08 Hz) fMRI data. Because temporal filtering influences the shape of the autocorrelation function, restricting the signal to slower BOLD fluctuations can produce a more slowly decaying ACF, making the ACF-sum measure particularly sensitive to longer-lag positive autocorrelation. In contrast, EEG, ECoG, and sEEG studies capture faster electrophysiological dynamics over broader frequency ranges. Thus, developmental decreases in intrinsic timescale reported in electrophysiological studies may reflect maturation-related changes in fast local electrophysiological dynamics, rather than the same slow BOLD processes captured by band-pass filtered fMRI. The association-cortex increases observed in our study could reflect increasing persistence of slow network-level BOLD dynamics across childhood and adolescence, rather than necessarily contradicting evidence for faster local neural dynamics with maturation. We emphasize that this interpretation remains speculative, and that future multimodal studies applying matched timescale definitions across fMRI and electrophysiology will be needed to directly test this possibility.

We have modified the Discussion section of the manuscript to reflect on this point (“Discussion” section, paragraph #6):

Furthermore, prior studies using imaging modalities with higher temporal resolution, such as EEG and ECoG, have reported overall decreases in intrinsic timescale during development (McKeon et al., 2025; Miles et al., 2026). Although similar measures of timescale were used across these studies and our study in fMRI, the overall age-related trends in timescale varied across modalities. These differences may reflect the fact that different imaging modalities probe distinct aspects of neural dynamics, as they are sensitive to different frequency ranges and may preferentially capture activity arising from different laminar sources (Scheeringa et al., 2016; Sadaghiani & Wirsich, 2020). In this context, developmental decreases in electrophysiological timescale and increases in fMRI-based ACF-sum timescale may not necessarily reflect opposing developmental patterns. Instead, electrophysiological studies may be more sensitive to faster local neural dynamics, whereas the present fMRI-based measure reflects the persistence of slow BOLD fluctuations within the 0.01-0.08 Hz range. Because temporal filtering influences the shape of the autocorrelation function (Shumway & Stoffer, 2017), restricting the signal to slower BOLD fluctuations can produce a more slowly decaying ACF. Consistent with this interpretation, our short- and long-lag decomposition of the fMRI autocorrelation function suggests that the ACF-sum measure is particularly sensitive to longer-lag positive autocorrelation, indicating that the association-cortex increases observed here should not be interpreted as a global slowing of neural activity across all temporal scales. Finally, discrepancies across studies, even within the same modality, may also arise from differences in data acquisition, preprocessing pipelines, and analytic approaches. Such methodological variation can influence estimates of resting-state dynamics (e.g., time series properties, cross-correlations, and intrinsic timescale; Li et al., 2024) and should be considered when interpreting findings.

*3. Comparing the authors own results in the manuscript, it appears that the independent adult sample had significantly shorter timescales (ranging from ~3.7-4.4 s) than either of the developmental samples (timescales ranging from ~4.6 - 5.5 s). I understand that the authors are more focused on the lack of age-dependent changes in the adult sample. Still, the fact that timescales were generally found to be lower - both globally and regionally - in the adult sample needs to be reconciled with the authors' claims that association region timescales increase throughout adolescence and remain stable in sensorimotor areas.*

Thank you for raising this important point. We agree that absolute timescale values in the young adult sample should be interpreted cautiously. Our primary developmental claims are based on within-dataset age effects in HCPD and HBN, rather than direct comparisons of absolute timescale values across developmental and adult datasets. Although the same timescale metric was used across datasets, all three cohorts differ in acquisition parameters and sample characteristics. Additionally, we note that the analysis was applied separately to each dataset without harmonizing them to ensure independent replication and specificity analysis. Therefore, we used the young adult dataset primarily to test the specificity of the findings and assess whether age-related differences in intrinsic timescale continues across young adulthood, rather than to infer absolute continuity from adolescence to adulthood. We have revised the manuscript to further clarify this point:

“Results” section, “Timescale remains relatively stable in adulthood” subsection:

Overall, these results emphasize the specificity of the developmental findings in HCPD and HBN to the developmental period of 8 to 22 years old, suggesting that fMRI intrinsic timescale develops along the S–A axis in youth and stabilizes in adulthood. Given the differences in acquisition parameters and cohort characteristics, the young adult dataset was used to assess the specificity of the developmental findings to the developmental cohorts and the stability of age-related effects within young adulthood, rather than as a direct continuation of the developmental age trajectories.

“Discussion” section, paragraph #1:

Moreover, findings in a young adult sample demonstrated that age-related differences in the intrinsic timescale are specific to the developmental cohorts and that the intrinsic timescale appears to remain stable in young adulthood.

*>Suggested analyses to test whether sensorimotor timescales continue diverging from association regions during middle childhood/adolescence:*

*A) Testing whether timescale differences between regions near opposing poles of the SA axis become increasingly larger with age than differences between regions at similarly situated points of the SA axis. In other words: are unimodal region timescales more like each other than they are to transmodal regions? Does the size of the difference between the unimodal-to-unimodal comparisons and the unimodal-to-transmodal comparisons depend on age? The null hypothesis here would be that the within and across hierarchy timescale comparisons do not change throughout adolescence. Age-dependent increases, however, would align with a model of increased SA timescale differentiation. This analysis would be especially impactful if done using within-subject SA axis comparisons, though would not necessarily help to establish whether any differences were due to sensorimotor timescale decreases, association timescale increases, or differences in relative rates of change.*

Furthermore, following the Reviewer’s suggestion, we performed two additional supplementary analyses in HCPD to test whether timescale differences between regions near opposing poles of the S-A axis become larger with age. First, we performed a within-subject distance-based analysis (**Figure S1a**). For each participant, we divided cortical regions into lower-ranking sensorimotor and higher-ranking association regions based on their position along the S-A axis. We then computed pairwise timescale differences within sensorimotor/association regions and across sensorimotor-to-association regions. For each participant, we compared across-hierarchy differences to within-hierarchy differences using a *t*-test, resulting in a subject-level estimate of timescale differentiation quantified by *t*-values. We then tested whether this measure of timescale differentiation across the hierarchy increased with age using Generalized Additive Models (GAMs). This analysis showed a significant positive association with age (**Figure S1a**; partial *R*^2^ = 0.025, *p*_anova_ = 0.0007), indicating that across-hierarchy timescale differences became larger relative to within-hierarchy differences across the HCPD age range. Statistical significance was assessed using the same GAM framework as our main analyses, comparing a full model including age as a smooth term to a reduced model containing only covariates.

Second, we performed a simpler complementary analysis in which we directly compared each participant’s regional timescale values in sensorimotor versus association cortex. Specifically, for each participant, we performed a *t*-test comparing timescale values between sensorimotor and association regions. We then modeled the resulting subject-level *t*-values as a function of age using a GAM. This analysis also showed a significant positive association with age (**Figure S1b**; partial *R*^2^ = 0.026, *p*_anova_ = 9.36 x 10^-5^), indicating that the difference between association and sensorimotor timescales increased across the HCPD age range. Statistical significance was assessed using the same GAM framework described above.

Together, these analyses provide evidence that the categorical separation between sensorimotor and association timescales increases with age in HCPD. We have therefore added this analysis as a supplementary figure and revised the Methods, Results, and Discussion sections.

In the Methods section, we have added:

**Sensorimotor-association timescale differentiation**

To test whether timescale differentiation between sensorimotor and association cortex increased with age, we performed two supplementary analyses in HCPD. First, we conducted a within-subject distance-based analysis. Cortical regions were divided into lower-ranking sensorimotor and higher-ranking association regions based on their position along the S-A axis. For each participant, we computed pairwise absolute timescale differences among regions within the same end of the hierarchy and across sensorimotor-to-association regions. We then compared across-hierarchy differences to within-hierarchy differences using a two-sample *t*-test, yielding a subject-level *t*-value indexing sensorimotor-association timescale differentiation. We then tested whether this differentiation measure varied with age using a GAM with the same model structure as the main analysis.

Second, we performed a complementary analysis in which regional timescale values were directly compared between sensorimotor and association cortex for each participant using a two-sample *t*-test. The resulting subject-level *t*-values were then modeled as a function of age using the same GAM framework. For both analyses, effect size was quantified using partial *R*^2^ and significance was assessed by comparing the full model including age to a reduced model containing only covariates.

In the Results section, we revised the “Neurodevelopmental patterns in intrinsic timescale align with the cortical hierarchy” subsection:

Furthermore, we found that regional neurodevelopmental patterns were heterogeneous across the cortex, with smaller age effects in the sensorimotor cortex and larger age effects in association cortex (**Figure 3b**). Notably, the effect size of the developmental associations aligned with the cortical hierarchy defined by the S–A axis (**Figure 3c**; *r*_s_ = 0.41, *p*_spin_ = 0.001). Consistent with this hierarchical pattern, supplementary within-subject analyses showed that the categorical separation between sensorimotor and association timescales increased with age (**Figure S1**). Further inspection of regional developmental model fits revealed that timescale increased in association cortex but was stable in sensorimotor regions (**Figure 3d**). Overall, these findings demonstrate that fMRI intrinsic timescale increases with age in youth, mainly driven by increases in timescale in association cortex. Timescale development reflected a hierarchical pattern, recapitulating the S–A axis of cortical organization.

And in the Discussion section, paragraph #2:

Our findings suggest that age-related differences in fMRI intrinsic timescale are broadly organized along the cortical hierarchy. Across youth, regional patterns of intrinsic timescale differences are aligned with the sensorimotor-to-association gradient that defines adult brain architecture. Additionally, supplementary within-subject analyses further suggest that the categorical separation between sensorimotor and association timescales also increases with age.

**Figure S1. Sensorimotor-association timescale differentiation in HCPD |** We tested whether timescale differentiation between sensorimotor and association regions increased with age. **(a)** In a within-subject distance-based analysis, cortical regions were divided into lower-ranking sensorimotor and higher-ranking association regions based on their position along the S-A axis. For each participant, pairwise timescale differences were computed within sensorimotor/association regions and across sensorimotor-to-association regions. Across-hierarchy differences were compared to within-hierarchy differences using a two-sample *t*-test, generating a subject-level estimate of timescale differentiation. This measure showed a significant positive association with age using a GAM (partial *R*^2^ = 0.025, *p*_anova_ = 0.0007). **(b)** In a complementary analysis, sensorimotor and association timescale values were directly compared within each participant using a two-sample *t*-test. The resulting subject-level *t*-values also showed a significant positive association with age using a GAM (partial *R*^2^ = 0.026, *p*_anova_ = 9.36 x 10^-5^). Each data point represents an individual participant. GAM fits are shown with black lines.

*B) Assessing correlations between age-of-peak-change and SA axis. The hypothesis that sensorimotor region timescales develop early and remain stable throughout development predicts lower ages-of-peak-change and smaller derivatives relative to association regions. This could be an addition to supplemental figure 1, which already calculates age-to-peak change, and would supplement the prior suggested analysis.*

Following the Reviewer’s suggestion, we assessed whether the estimated age of peak change in intrinsic timescale was associated with position along the S-A axis. In our analysis, age of peak change was derived from the first derivative of each region’s GAM smooth function. Specifically, for each region, we identified the age at which the significant derivative reached its maximum absolute value, reflecting the age at which the fitted age trajectory showed the strongest rate of change. We then correlated these age-of-peak-change estimates with regional S-A axis ranks. This analysis did not show a significant association in either developmental dataset (HCPD: *r*_s_ = 0.06, *p*_spin_ = 0.74; HBN: *r*_s_ = -0.19, *p*_spin_ = 0.14).

However, we interpret these correlations cautiously because age of peak change is only defined for regions with at least one significant derivative. Regions with weak, stable, or uncertain age trajectories do not have a well-defined age of peak change and were therefore assigned missing values. As a result, the correlation analysis included only 211 of 400 regions in HCPD and 144 of 400 regions in HBN. Thus, this test does not provide a whole-cortex assessment of whether age-related timescale differences align with the S-A axis, but rather evaluates this association only among regions with reliably detected derivative-based change.

To address the Reviewer’s broader question, we added a complementary analysis estimating the age at which regional rates of timescale differences were maximally aligned with the S-A axis (Sydnor et al., 2023). Specifically, we used the first derivative of each region’s GAM smooth function to estimate the age-specific rate of timescale differences (i.e., slope of developmental differences). We then quantified, at each age, the spatial correlation between regional derivatives and S-A axis rank. This approach does not require assigning a single age of peak change to each region and instead asks when the whole-cortex pattern of age-related timescale differences is most aligned with the cortical hierarchy. This analysis showed that alignment between age-related timescale differences and the S-A axis peaked in early-to-mid adolescence in both developmental datasets, with maximal alignment at 14.6 years in HCPD and 12.8 years in HBN (**Figure S2b**).

We have now added this analysis to the revised supplementary **Figure S2** (previously **Figure S1**) and updated the manuscript to clarify that age-of-peak-change estimates are informative but should be interpreted cautiously when region-wise GAM derivatives are not significant across all regions.

“Results” section, “Findings replicate in an independent developmental cohort” subsection:

For regions with at least one significant derivative, we summarized the timing of maximal development as the age at which the regional model derivative reached its maximum absolute value (**Figure S2a**). These estimates should be interpreted cautiously because age of maximal development is only defined for regions with significant derivative-based change; regions with weak, stable, or uncertain age trajectories do not have a well-defined peak-change age. To complement this analysis, we also examined the age at which whole-cortex rates of timescale differences were maximally aligned with the S-A axis, which peaked in early-to-mid adolescence in both datasets (**Figure S2b**; 14.6 years in HCPD and 12.8 years in HBN).

“Methods” section:

**Age of maximum alignment between age-related timescale differences and the S-A axis**

To assess when age-related differences in intrinsic timescale were most strongly aligned with the cortical hierarchy, we estimated age-specific rates of timescale differences from the first derivative of each region’s GAM smooth function for age (Sydnor et al., 2023). These derivatives represent the estimated rate of timescale differences per one-year age interval. For each age from 8 to 22 years, we quantified the spatial association between regional derivative values and regional S-A axis ranks using Spearman’s rank correlation. To account for uncertainty in derivative estimates, we sampled 10,000 draws from the posterior distribution of the derivative for each regional GAM smooth and computed the age-specific correlation between derivative values and S-A axis ranks for each draw. The median correlation across draws was used to summarize the age-specific alignment between timescale differences and the S-A axis, and the 95% credible interval was calculated from the distribution of sampled correlations. We then identified the age of maximum alignment as the age at which the derivative-to-S-A axis correlation was maximal for each posterior draw. The median and 95% credible interval of these ages were used to summarize the timing of maximum alignment between age-related timescale differences and the S-A axis.

**Figure S2. Timing of age-related differences in intrinsic timescale across two developmental cohorts |** **(a)** Derivatives of the intrinsic timescale regional developmental patterns (**Figure 3d** and **Figure 4d**) were calculated as finite differences between consecutive time points to estimate the age at which timescale development demonstrated maximal change. Specifically, the age of peak change was estimated as the age where the derivative was greatest in absolute magnitude. **(b)** To estimate the age at which age-related timescale differences were maximally aligned with the S-A axis, we calculated the first derivative of each region’s GAM smooth function for age. These derivatives represent the estimated rate of timescale differences per one-year age interval. At each age, we quantified the spatial correlation between regional derivative values and regional S-A axis ranks. To account for uncertainty in derivative estimates, we sampled 10,000 draws from the posterior derivative distribution and calculated the derivative-to-SA axis correlation for each draw. The black line shows the median correlation across draws, and the gray band shows the 95% credible interval. The pink band shows the 95% credible interval for the age of maximum alignment. Maximal alignment occurred at 14.6 years in HCPD and 12.8 years in HBN.

*---*

*While the specific analyses suggested should be thought of as recommendations, not requirements, further effort to clarify the relationship between the authors' results and the current state-of-the-field will be essential to support their conclusions.*

*I look forward to seeing how the manuscript develops in revision and commend the authors for a thoughtful and timely submission.*

We thank the Reviewer for their thorough and helpful feedback!

## Reviewer #2 (Hoyt Patrick Taylor IV):

*The authors carry out an analysis of intrinsic timescale in fMRI using the HCPD, HBN, and HCPYA cohorts. They find that development of intrinsic timescale as measured using ACF plays out along the S-A axis during youth, driven primarily by increasing timescales in association cortex.*

*The replication analysis and the inclusion of the HCPYA cohort are clear strengths. The authors candidly engage with a recent paper (Bero et al. 2026) which also analyzed timescale in the HCPD cohort. The findings of Bero et al. 2026 are at odds with the findings of this work, which I believe requires more rigorous treatment from the authors.*

*This manuscript reports stable intrinsic timescale in sensorimotor cortex and increases in association cortex, while Bero et al. reports decreases in sensorimotor and increases in prefrontal cortex. Authors propose that this discrepancy is accounted for by the fact that the full ACF captures different dynamics than lag-1 TA, however I think the manuscript would benefit from further analyzing this hypothesis.*

*I would encourage the authors to compute lag-1 TA on the same preprocessed HCPD data and demonstrate the relationship to their ACF-sum metric, verifying the Bero et al finding of decreasing lag-1 TA in sensorimotor cortex. If the ACF sum could be decomposed into short-lag and longer-lag contributions, this could potentially reveal the mechanism for the discrepancy between these metrics.*

*If the authors opt not to carry out the above analysis, the discussion should at least be expanded to acknowledge that the disagreement is not mechanistically understood and to outline what would be necessary to probe the mechanism empirically.*

*Outside of this concern, I am convinced by the conclusions of the paper.*

We thank the Reviewer for these very helpful suggestions. Following the Reviewer’s comments, we have performed two supplementary analyses to compare and contextualize our findings more thoroughly with respect to the recent paper by Bero et al. (2026). The two analyses are as following:

Intrinsic timescale estimated using lag-1 autocorrelation:

Following the same approach as used by Bero et al. (2026), we estimated intrinsic timescale using lag-1 temporal autocorrelation for each brain region and participant (i.e., lag-1 TA). This allowed us to directly compare the ACF-sum timescale metric (i.e., our original measure) with lag-1 TA within the same sample, preprocessing pipeline, parcellation, and statistical framework. We found that the mean intrinsic timescale maps measured by our approach and the Bero et al. approach were not significantly correlated (**Figure S10a**; *r*_s_ = 0.17, *p*_spin_ = 0.23). Repeating the developmental analysis, we found a trend-like positive association between the whole-brain average lag-1 TA and participant age (**Figure S9a**). Given that our main focus was on regional developmental patterns, we also repeated our regional analysis using lag-1 TA. At the regional level, lag-1 TA showed both age-related increases and decreases across cortex (**Figure S9b**), with age-related increases most prominently visible in frontal/prefrontal regions, partially consistent with Bero et al. Overall, the regional age effects estimated using the two metrics followed a similar pattern (**Figure S10b**; *r*_s_ = 0.55, *p*_spin_ = 0.0001). However, the age effects estimated from lag-1 TA did not follow the S-A axis, as we had demonstrated in our original analysis (**Figure S9c**; *r*_s_ = -0.005, *p*_spin_ = 0.9). Thus, lag-1 TA and ACF-sum timescale show partially overlapping developmental effects.

Overall, our findings with lag-1 TA partially confirm the findings from Bero et al. (2026). Specifically, we find a similar increase in lag-1 TA in prefrontal regions as reported by Bero et al. The increase in prefrontal regions was also present in our original analysis using the full autocorrelation function to estimate intrinsic timescale (i.e., ACF-sum timescale metric). However, we still observed a trend-like increase in the whole-brain average timescale with age even when we used lag-1 TA. We speculate that these remaining discrepancies might partially be due to several sources related to preprocessing, methodology, and sample selection procedure. A few of those are outlined below:

- *Differences in sample selection.* Bero et al. included participants within the age range of 5-22 years old. They also used a mean framewise displacement (FD) threshold of 0.34 mm as their motion exclusion criterion during sample selection (total *n* = 627). We included participants within the age range of 8-22 years old, to align our sample with HBN data, and applied a stricter mean FD threshold of 0.2 mm (total *n* = 565).
- *Differences in preprocessing.* Bero et al. used the minimally processed HCPD data with ICA-FIX denoising and high-pass filtering at 0.0005 Hz. They then applied a high-pass filter at 0.008 Hz to the parcellated time series. We preprocessed the raw HCPD data using fMRIPrep and then post-processed them with XCP-D, applying a band-pass filter at 0.01-0.08 Hz to the preprocessed time-series data. Because lag-1 TA is sensitive to short-lag smoothness, differences in temporal filtering may influence the amount of high-frequency variance retained in the BOLD signal and therefore affect the metric and its age-related patterns.
- *Methods used to assess age effects.* Bero et al. used linear regression models to generate brain maps of age effects. We used generalized additive models (GAMs) to assess linear and nonlinear age effects.

Although the partially overlapping findings are promising despite these differences, a more systematic direct comparison of the two measures would only be feasible using the exact same processing pipelines (e.g., using the minimally processed HCPD data). However, even within our own pipeline, lag-1 TA and ACF-sum timescale appear to capture partially distinct aspects of temporal autocorrelation. Following the Reviewer’s extremely helpful suggestion, we performed an additional analysis decomposing the ACF-sum metric into short- and long-lag contributions (as described below).

Decomposition of intrinsic timescale into short- vs long-lag contributions:

Next, as suggested, we investigated whether the discrepancy between lag-1 TA and ACF-sum timescale reflects different contributions from early versus later portions of the ACF. To do this, we first computed a group-average ACF across all parcels and participants and identified a data-driven elbow lag, ***k***, using a two-piece linear fit to the positive portion of the group-average ACF. We searched candidate elbow lags from ***k*** = 3 to 47 within the positive group-average ACF, corresponding to 2.4-37.6 s. The two-piece linear fit identified an elbow at lag ***k*** = 5, corresponding to 4.0 s. We then used this same lag ***k*** for all parcels and participants to decompose the original ACF-sum timescale into a short-lag component, Int_short_ , defined as the ACF sum from lag 0 through ***k***, and a long-lag component, Int_long_ , defined as the ACF sum after ***k*** and up to the first zero crossing.

This analysis revealed that the mean lag-1 TA (regional mean across-subjects) was almost perfectly associated with the mean short-lag component of the ACF-sum timescale (**Figure S11b**; *r*_s_ = 0.98, *p*_spin_ = 0.0001), but was not significantly associated with the mean long-lag component (**Figure S11d**; *r*_s_ = -0.26, *p*_spin_ = 0.16). In contrast, the original ACF-sum timescale was strongly associated with the long-lag component (**Figure S11c**; *r*_s_ = 0.86, *p*_spin_ = 0.0001), and only modestly associated with the short-lag component (**Figure S11a**; *r*_s_ = 0.31, *p*_spin_ = 0.01).

Importantly, we observed a similar pattern when comparing the spatial maps of age effects. Age effects estimated using lag-1 TA were highly correlated with age effects for Int_short_ (**Figure S11b**; *r*_s_ = 0.93, *p*_spin_ = 0.0001), but only weakly-to-moderately correlated with age effects for Int_long_ (**Figure S11d**; *r*_s_ = 0.36, *p*_spin_ = 0.0002). Conversely, age effects estimated using the original ACF-sum timescale were more strongly correlated with age effects for Int_long_ (**Figure S11c**; *r*_s_ = 0.96, *p*_spin_ = 0.0001) than with age effects for Int_short_ (**Figure S11a**; *r*_s_ = 0.71, *p*_spin_ = 0.0001).

Together, these findings suggest that the discrepancy between lag-1 TA and ACF-sum timescale may reflect differences in their sensitivity to the shape of the ACF. Lag-1 TA captures the immediate rate of decorrelation at lag 1, whereas the ACF-sum measure is sensitive to broader features of the ACF curve, including longer positive tails across later lags. This distinction was also evident in the developmental effects, suggesting that short- and long-lag ACF components capture partially distinct temporal properties and developmental patterns.

We have now made several modifications to the paper to reflect these findings:

“Methods” section:

**Intrinsic timescale estimated using lag-1 temporal autocorrelation**

As a complementary estimate of intrinsic timescale, we calculated lag-1 temporal autocorrelation (lag-1 TA) for each brain region and participant, following the approach used by Shinn et al. (2023) and Bero et al. (2026). Lag-1 TA quantifies the similarity between a time series and a copy of itself shifted by one time point. In the context of fMRI, this corresponds to the correlation between the BOLD signal at time point ***t*** and the BOLD signal at the immediately following time point, ***t***+1 , within the same brain region. Higher lag-1 TA values therefore indicate slower decorrelation, or greater temporal persistence, across consecutive time points, whereas lower values indicate faster decorrelation from one time point to the next.

Fully processed parcellated fMRI time series were first z-score normalized within each parcel. We then estimated lag-1 TA using the temporal_autocorrelation(x) function from the “spatiotemporal” Python package (Shinn et al., 2023). This function computes TA-𝚫_1_ , defined as the first-order temporal autocorrelation between consecutive time points. For each participant, the input was a parcel-by-time matrix, and the output was a vector of lag-1 TA values across parcels. Unlike the ACF-sum timescale measure, lag-1 TA captures *only* the first nonzero lag of the ACF rather than the area under the positive portion of the ACF. Accordingly, lag-1 TA was not integrated across multiple lags and was not multiplied by TR; it is a dimensionless autocorrelation coefficient. We repeated subsequent analyses using these parcellated lag-1 TA maps to compare regional developmental patterns with those estimated using the original ACF-sum timescale metric.

**Short- and long-lag contributions to intrinsic timescale**

To examine whether the ACF-sum measure of intrinsic timescale was differentially driven by short- versus longer-lag autocorrelation structure, we decomposed the positive ACF area into short- and long-lag components. First, using the normalized parcellated fMRI time series, we estimated the ACF for each parcel and participant as described above. We then computed a group-average ACF by averaging ACF curves across all parcels and participants. To identify a data-driven lag separating the steep early decay of the ACF from the slower positive tail, we fit a two-piece linear model to the positive portion of the group-average ACF. Specifically, for each candidate lag ***k***, we fit one linear function to the early-lag portion of the group-average ACF and a second linear function to the later-lag portion. We selected the lag ***k*** that minimized the total sum of squared errors across the two fitted line segments. Lag 0 was excluded from this elbow estimation because the ACF at lag 0 is fixed at 1. We searched candidate elbow lags from ***k*** = 3 to 47 within the positive group-average ACF, corresponding to 2.4-37.6 s in HCPD (TR = 0.8 s). The two-piece linear fit identified an elbow at lag ***k*** = 5, corresponding to 4.0 s in HCPD.

Using this single group-level elbow lag ***k***, we then returned to the participant- and parcel-level ACFs and calculated two complementary timescale estimates. The short-lag component, Int_short_ , was calculated as the sum of ACF values from lag 0 through lag ***k***, bounded by the first zero-crossing point when the ACF crossed zero before lag ***k***. The long-lag component, Int_long_ , was calculated as the sum of positive ACF values after lag ***k*** and up to the first zero-crossing point. Both components were multiplied by the dataset-specific TR to express them in seconds. This decomposition ensured that Int_short_ and Int_long_ summed to yield the original positive ACF-sum intrinsic timescale estimate for each parcel and participant. We used this approach to test whether short- and long-lag ACF components captured distinct aspects of development, and specifically whether lag-1 autocorrelation preferentially reflected the short-lag component of the ACF.

“Results” section, “Sensitivity analysis” subsection, paragraph #2:

To test whether intrinsic timescale estimates were sensitive to methodological choices and to better contextualize our findings with prior work, we compared our ACF-sum measure with several alternative approaches for quantifying intrinsic timescale in HCPD. First, we quantified intrinsic timescale using two other approaches based on full ACF: (1) fitting an exponential function to ACF and using the exponential decay constant to estimate intrinsic timescale (Ito et al., 2020); (2) directly using the first zero-crossing point of ACF as intrinsic timescale. Both approaches generated intrinsic timescale maps and developmental patterns consistent with the original method (**Figure S8**). We further repeated the HCPD analyses using lag-1 temporal autocorrelation (lag-1 TA), following prior work (Shinn et al., 2023; Bero et al., 2026). We found that lag-1 TA and ACF-sum timescale differed in their mean spatial distribution but showed partially overlapping developmental effects (**Figures S9-S10**). We also decomposed the ACF-sum timescale into short- and long-lag components and found that lag-1 TA was most closely related to short-lag autocorrelation, whereas the full ACF-sum measure was more strongly influenced by longer-lag positive autocorrelation (**Figure S11**). Together, these analyses suggest that our core findings are robust across several full-ACF-based timescale definitions, while also showing that different metrics emphasize different features of temporal persistence: lag-1 TA primarily reflects short-lag autocorrelation, whereas the ACF-sum measure is more sensitive to longer-lag positive autocorrelation across the full ACF. This suggests that the developmental effects observed with our ACF-sum measure were driven primarily by long-lag ACF structure.

“Discussion” section, new paragraph #4:

To further investigate these differences, we repeated our developmental analyses using lag-1 TA in the same HCPD sample and preprocessing pipeline used for our primary ACF-sum analyses. This comparison showed that lag-1 TA and ACF-sum timescale had distinct regional mean maps but demonstrated partially overlapping regional age effects. Decomposing the ACF-sum timescale into short- and long-lag components further suggested that lag-1 TA and ACF-sum timescale emphasize partially distinct features of the ACF: lag-1 TA primarily reflects immediate short-lag decorrelation, whereas ACF-sum timescale is more sensitive to the broader shape of the ACF, including sustained positive autocorrelation across longer lags. Thus, while methodological differences in preprocessing, sample selection, and modeling may contribute to discrepancies across studies, our results indicate that some differences may also arise because commonly used timescale metrics emphasize different characteristics of the same fMRI signal.

To more explicitly reflect these new findings, we also modified the following sentence in paragraph #3 of the “Discussion” section:

… Despite these differences in how fMRI dynamics were defined, both studies converge in showing that the development of neural dynamics is spatially heterogeneous across cortex and broadly related to large-scale cortical hierarchy. Additionally, the regional heterogeneity of age-related differences observed across both measures suggests that they capture partially overlapping but distinct aspects of neural dynamics. For example, we found that intrinsic timescales remain stable in sensorimotor regions while they increase in association cortex, whereas Bero et al. found that temporal scales decrease in sensorimotor regions while they increase in prefrontal regions.

**Figure S9. Developmental patterns of lag-1 temporal autocorrelation in HCPD |** Lag-1 temporal autocorrelation (lag-1 TA) was estimated in HCPD as a complementary measure of intrinsic timescale. **(a)** Cortical map of lag-1 TA and relationship between whole-brain average lag-1 TA and age. Each data point in the scatter plot represents an individual participant (partial *R*^2^ = 0.008, *p*_anova_ = 0.03). **(b)** Region-wise GAMs identified heterogeneous age effects (i.e., partial *R*^2^) across cortex. FDR-corrected maps show regions with significant age effects (*p*_FDR_ < 0.05; 28.2% of regions with significant age effects). **(c)** Age effects on lag-1 TA were not significantly associated with the S-A axis. Significance of the association between age effects and S-A axis rank was assessed using 10,000 spin tests. *r*_s_ denotes Spearman’s rank correlation coefficient. Linear regression line is added for visualization purposes only. **(d)** Regional trajectories of developmental patterns of lag-1 TA were obtained from region-wise GAM results. Each line corresponds to the model fit of each cortical region and is colored based on the region’s rank along the S-A axis.

**Figure S10. Relationship between ACF-sum intrinsic timescale and lag-1 temporal autocorrelation |** We compared regional maps of intrinsic timescale estimated using the original ACF-sum method and lag-1 temporal autocorrelation (lag-1 TA) in HCPD. **(a)** Regional intrinsic timescale maps estimated using the two approaches were not significantly correlated. **(b)** In contrast, regional age effects (i.e., partial *R*^2^) estimated using the two approaches were significantly correlated. Each data point in the scatter plot represents a brain region from the Schaefer 400 atlas. The data points are colored based on their rankings on the S-A axis. *r*_s_ denotes Spearman’s rank correlation coefficient. Significance was assessed using 10,000 spin tests. Linear regression lines are added for visualization purposes only.

**Figure S11. Short- and long-lag contributions to ACF-sum intrinsic timescale |** To examine whether ACF-sum intrinsic timescale was differentially driven by short- versus longer-lag autocorrelation structure, we decomposed the positive ACF area into short-lag and long-lag components. **(a)** The short-lag component was modestly associated with the intrinsic timescale estimated using the original ACF-sum method. The regional age effects from the short-lag component were moderately associated with age effects from the original method. **(b)** In contrast, the short-lag component was highly associated with lag-1 temporal autocorrelation (lag-1 TA), both for regional timescale maps and regional age effects. **(c)** The long-lag component and its age effects were strongly associated with the original findings estimated using the ACF-sum method. **(d)** In contrast, the long-lag component was not significantly associated with lag-1 TA in regional timescale maps and was only weakly associated with lag-1 TA regional age effects. Each data point in the scatter plot represents a brain region from the Schaefer 400 atlas. The data points are colored based on their rankings on the S-A axis. *r*_s_ denotes Spearman’s rank correlation coefficient. Significance was assessed using 10,000 spin tests. Linear regression lines are added for visualization purposes only.

## Reviewer #3:

*The manuscript addresses an interesting topic by characterizing the spatial distribution and developmental trajectories of intrinsic neural timescales (INT). However, I have several concerns regarding the framing, methodological justification, and strength of the conclusions.*

*1. The rationale that previous reports of lifespan changes in neural dynamics (Bero et al., 2026) motivate the present analysis also requires further clarification, particularly the manuscript does not offer a clear conceptual and methodological advance beyond prior work.*

We agree that the rationale and conceptual advance of the present study could be stated more clearly. We have now expanded the Introduction to clarify that intrinsic timescale can be quantified using several approaches, including lag-1 temporal autocorrelation, ACF decay time to a fixed threshold, exponential decay constants, spectral parameterization, and the sum of the positive portion of the ACF. We now emphasize that the ACF-sum measure is widely used in fMRI studies of intrinsic timescale, but that its cortical organization and age-related patterns in youth remain poorly characterized. Thus, applying this metric across large developmental fMRI cohorts addresses an important gap while providing a complementary perspective to lag-1 TA and other timescale definitions.

We also clarified why ACF-sum may be informative beyond single-lag or fixed-threshold measures. Specifically, ACF-sum incorporates autocorrelation structure across multiple positive lags, rather than relying on a single lag estimate or a fixed decay point. This allows the measure to capture both short-lag autocorrelation and the longer positive tail of the ACF. Consistent with this rationale, our new analyses included in this revision showed that lag-1 TA primarily indexes short-lag persistence, whereas ACF-sum timescale is more strongly influenced by longer-lag positive autocorrelation (see our response to the next comment).

In response to the Reviewer’s comment, we have modified the Introduction in the revised manuscript.

“Introduction” section, new paragraph #4:

Intrinsic timescale can be quantified in several ways, including lag-1 temporal autocorrelation, decay time to a fixed threshold of the autocorrelation function (ACF), exponential decay constants, spectral parameterization, and the sum of the positive portion of the ACF (Gao et al., 2020; Raut et al., 2020; Shinn et al., 2023; Bero et al., 2026). Here, we focus on the ACF-sum measure because it is a widely used approach in fMRI studies of intrinsic timescale and incorporates autocorrelation structure across multiple positive lags, rather than relying on a single lag estimate or fixed threshold. This provides a complementary perspective on temporal persistence by capturing both short-lag autocorrelation and the longer positive tail of the ACF. Despite its use in fMRI studies of neural dynamics, relatively little is known about how ACF-sum intrinsic timescale varies across the cortex during youth. Applying this measure in large developmental fMRI datasets therefore fills an important gap and enables comparison with prior fMRI studies using similar full-ACF approaches.

“Introduction” section, modified paragraph #7:

In this study, we map developmental patterns of intrinsic timescale across childhood and adolescence using resting-state fMRI from a large cohort of typically developing youth. By applying an ACF-sum measure of intrinsic timescale, we characterize temporal persistence across the positive portion of the ACF and evaluate how these regional patterns relate to the cortical hierarchy defined by the S-A axis. We further assess the generalizability of the observed developmental patterns in a large independent developmental sample. We then assess the specificity of our developmental results by also examining associations between intrinsic timescale and age in young adulthood. Together, these analyses clarify how fMRI-based intrinsic timescale varies across youth and how these age-related differences relate to large-scale cortical organization.

*2. The rationale for using a full-shape autocorrelation-based INT measure should be clarified. Alternative definitions, including ACF decay time to a fixed threshold(0.5 or 1/e) (Raut et al. 2020; Gao et al. 2020), lag-1 temporal autocorrelation (Bero et al. 2026) and spectral parametrization(Gao et al. 2020), are widely used. Since the authors acknowledge in the Discussion that different INT definitions may capture partially overlapping but distinct aspects of neural dynamics, it would be valuable to examine the extent to which the main spatial and developmental findings converge or diverge across alternative INT metrics within the same analytic framework.*

We thank the Reviewer for this important suggestion. We agree that the rationale for using the full positive ACF-sum measure of intrinsic timescale should be clarified, especially because multiple alternative definitions of intrinsic timescale are commonly used in the literature and may capture partially overlapping but distinct aspects of neural dynamics. We have now further clarified this in the revised Introduction (see our response to the previous comment).

In the original submission, we included a methodological sensitivity analysis comparing our ACF-sum measure with two alternative ACF-based measures: the first zero-crossing point of the ACF and the exponential decay constant from an exponential fit to the ACF. These analyses showed that both alternative ACF-based measures generated intrinsic timescale maps and developmental patterns that were broadly consistent with our original ACF-sum method (**Figure S8**). Thus, our main findings were not specific to the particular choice of summing the positive portion of the ACF.

Following the Reviewer’s comment, we have now expanded this comparison by estimating intrinsic timescale using lag-1 temporal autocorrelation (lag-1 TA), following the approach used by Shinn et al. (2023) and Bero et al. (2026). We repeated the full HCPD regional analysis using lag-1 TA. We found that the mean intrinsic timescale maps measured by our approach and the Bero et al. approach were not significantly correlated (**Figure S10a**; *r*_s_ = 0.17, *p*_spin_ = 0.23). Repeating the developmental analysis, we found a trend-like positive association between the whole-brain average lag-1 TA and participant age (**Figure S9a**). Given that our main focus was on regional developmental patterns, we also repeated our regional analysis using lag-1 TA. At the regional level, lag-1 TA showed spatially heterogeneous age effects, with both regional increases and decreases observed across cortex (**Figure S9b**). Overall, the regional age effects estimated using lag-1 TA and ACF-sum were moderately correlated (**Figure S10b**; *r*_s_ = 0.55, *p*_spin_ = 0.0001), indicating partially overlapping age-related spatial patterns.

To further understand why these metrics may diverge, we decomposed the ACF-sum timescale into short- and long-lag components. This analysis showed that lag-1 TA was almost perfectly associated with the short-lag component of the ACF-sum timescale (**Figure S11b**; *r*_s_ = 0.98, *p*_spin_ = 0.0001), but was not significantly associated with the long-lag component (**Figure S11d**; *r*_s_ = -0.26, *p*_spin_ = 0.16). In contrast, the original ACF-sum timescale was strongly associated with the long-lag component (**Figure S11c**; *r*_s_ = 0.86, *p*_spin_ = 0.0001), and only modestly associated with the short-lag component (**Figure S11a**; *r*_s_ = 0.31, *p*_spin_ = 0.01). Similar patterns were observed for regional age effects.

Together, these findings suggest that the discrepancy between lag-1 TA and ACF-sum timescale may reflect differences in their sensitivity to the shape of the ACF. Lag-1 TA captures the immediate rate of decorrelation at lag 1, whereas the ACF-sum measure is sensitive to broader features of the ACF curve, including longer positive tails across later lags. This distinction was also evident in the developmental effects, suggesting that short- and long-lag ACF components capture partially distinct temporal properties and developmental patterns.

We have now made several modifications to the manuscript to present these additional sensitivity analyses.

“Methods” section:

**Intrinsic timescale estimated using lag-1 temporal autocorrelation**

As a complementary estimate of intrinsic timescale, we calculated lag-1 temporal autocorrelation (lag-1 TA) for each brain region and participant, following the approach used by Shinn et al. (2023) and Bero et al. (2026). Lag-1 TA quantifies the similarity between a time series and a copy of itself shifted by one time point. In the context of fMRI, this corresponds to the correlation between the BOLD signal at time point ***t*** and the BOLD signal at the immediately following time point, ***t***+1 , within the same brain region. Higher lag-1 TA values therefore indicate slower decorrelation, or greater temporal persistence, across consecutive time points, whereas lower values indicate faster decorrelation from one time point to the next.

Fully processed parcellated fMRI time series were first z-score normalized within each parcel. We then estimated lag-1 TA using the temporal_autocorrelation(x) function from the “spatiotemporal” Python package (Shinn et al., 2023). This function computes TA-𝚫_1_ , defined as the first-order temporal autocorrelation between consecutive time points. For each participant, the input was a parcel-by-time matrix, and the output was a vector of lag-1 TA values across parcels. Unlike the ACF-sum timescale measure, lag-1 TA captures *only* the first nonzero lag of the ACF rather than the area under the positive portion of the ACF. Accordingly, lag-1 TA was not integrated across multiple lags and was not multiplied by TR; it is a dimensionless autocorrelation coefficient. We repeated subsequent analyses using these parcellated lag-1 TA maps to compare regional developmental patterns with those estimated using the original ACF-sum timescale metric.

**Short- and long-lag contributions to intrinsic timescale**

To examine whether the ACF-sum measure of intrinsic timescale was differentially driven by short- versus longer-lag autocorrelation structure, we decomposed the positive ACF area into short- and long-lag components. First, using the normalized parcellated fMRI time series, we estimated the ACF for each parcel and participant as described above. We then computed a group-average ACF by averaging ACF curves across all parcels and participants. To identify a data-driven lag separating the steep early decay of the ACF from the slower positive tail, we fit a two-piece linear model to the positive portion of the group-average ACF. Specifically, for each candidate lag ***k***, we fit one linear function to the early-lag portion of the group-average ACF and a second linear function to the later-lag portion. We selected the lag ***k*** that minimized the total sum of squared errors across the two fitted line segments. Lag 0 was excluded from this elbow estimation because the ACF at lag 0 is fixed at 1. We searched candidate elbow lags from ***k*** = 3 to 47 within the positive group-average ACF, corresponding to 2.4-37.6 s in HCPD (TR = 0.8 s). The two-piece linear fit identified an elbow at lag ***k*** = 5, corresponding to 4.0 s in HCPD.

Using this single group-level elbow lag ***k***, we then returned to the participant- and parcel-level ACFs and calculated two complementary timescale estimates. The short-lag component, Int_short_ , was calculated as the sum of ACF values from lag 0 through lag ***k***, bounded by the first zero-crossing point when the ACF crossed zero before lag ***k***. The long-lag component, Int_long_ , was calculated as the sum of positive ACF values after lag ***k*** and up to the first zero-crossing point. Both components were multiplied by the dataset-specific TR to express them in seconds. This decomposition ensured that Int_short_ and Int_long_ summed to yield the original positive ACF-sum intrinsic timescale estimate for each parcel and participant. We used this approach to test whether short- and long-lag ACF components captured distinct aspects of development, and specifically whether lag-1 autocorrelation preferentially reflected the short-lag component of the ACF.

“Results” section, “Sensitivity analysis” subsection, paragraph #2:

To test whether intrinsic timescale estimates were sensitive to methodological choices and to better contextualize our findings with prior work, we compared our ACF-sum measure with several alternative approaches for quantifying intrinsic timescale in HCPD. First, we quantified intrinsic timescale using two other approaches based on full ACF: (1) fitting an exponential function to ACF and using the exponential decay constant to estimate intrinsic timescale (Ito et al., 2020); (2) directly using the first zero-crossing point of ACF as intrinsic timescale. Both approaches generated intrinsic timescale maps and developmental patterns consistent with the original method (**Figure S8**). We further repeated the HCPD analyses using lag-1 temporal autocorrelation (lag-1 TA), following prior work (Shinn et al., 2023; Bero et al., 2026). We found that lag-1 TA and ACF-sum timescale differed in their mean spatial distribution but showed partially overlapping developmental effects (**Figures S9-S10**). We also decomposed the ACF-sum timescale into short- and long-lag components and found that lag-1 TA was most closely related to short-lag autocorrelation, whereas the full ACF-sum measure was more strongly influenced by longer-lag positive autocorrelation (**Figure S11**). Together, these analyses suggest that our core findings are robust across several full-ACF-based timescale definitions, while also showing that different metrics emphasize different features of temporal persistence: lag-1 TA primarily reflects short-lag autocorrelation, whereas the ACF-sum measure is more sensitive to longer-lag positive autocorrelation across the full ACF. This suggests that the developmental effects observed with our ACF-sum measure were driven primarily by long-lag ACF structure.

“Discussion” section, new paragraph #4:

To further investigate these differences, we repeated our developmental analyses using lag-1 TA in the same HCPD sample and preprocessing pipeline used for our primary ACF-sum analyses. This comparison showed that lag-1 TA and ACF-sum timescale had distinct regional mean maps but demonstrated partially overlapping regional age effects. Decomposing the ACF-sum timescale into short- and long-lag components further suggested that lag-1 TA and ACF-sum timescale emphasize partially distinct features of the ACF: lag-1 TA primarily reflects immediate short-lag decorrelation, whereas ACF-sum timescale is more sensitive to the broader shape of the ACF, including sustained positive autocorrelation across longer lags. Thus, while methodological differences in preprocessing, sample selection, and modeling may contribute to discrepancies across studies, our results indicate that some differences may also arise because commonly used timescale metrics emphasize different characteristics of the same fMRI signal.

**Figure S9. Developmental patterns of lag-1 temporal autocorrelation in HCPD |** Lag-1 temporal autocorrelation (lag-1 TA) was estimated in HCPD as a complementary measure of intrinsic timescale. **(a)** Cortical map of lag-1 TA and relationship between whole-brain average lag-1 TA and age. Each data point in the scatter plot represents an individual participant (partial *R*^2^ = 0.008, *p*_anova_ = 0.03). **(b)** Region-wise GAMs identified heterogeneous age effects (i.e., partial *R*^2^) across cortex. FDR-corrected maps show regions with significant age effects (*p*_FDR_ < 0.05; 28.2% of regions with significant age effects). **(c)** Age effects on lag-1 TA were not significantly associated with the S-A axis. Significance of the association between age effects and S-A axis rank was assessed using 10,000 spin tests. *r*_s_ denotes Spearman’s rank correlation coefficient. Linear regression line is added for visualization purposes only. **(d)** Regional trajectories of developmental patterns of lag-1 TA were obtained from region-wise GAM results. Each line corresponds to the model fit of each cortical region and is colored based on the region’s rank along the S-A axis.

**Figure S10. Relationship between ACF-sum intrinsic timescale and lag-1 temporal autocorrelation |** We compared regional maps of intrinsic timescale estimated using the original ACF-sum method and lag-1 temporal autocorrelation (lag-1 TA) in HCPD. **(a)** Regional intrinsic timescale maps estimated using the two approaches were not significantly correlated. **(b)** In contrast, regional age effects (i.e., partial *R*^2^) estimated using the two approaches were significantly correlated. Each data point in the scatter plot represents a brain region from the Schaefer 400 atlas. The data points are colored based on their rankings on the S-A axis. *r*_s_ denotes Spearman’s rank correlation coefficient. Significance was assessed using 10,000 spin tests. Linear regression lines are added for visualization purposes only.

**Figure S11. Short- and long-lag contributions to ACF-sum intrinsic timescale |** To examine whether ACF-sum intrinsic timescale was differentially driven by short- versus longer-lag autocorrelation structure, we decomposed the positive ACF area into short-lag and long-lag components. **(a)** The short-lag component was modestly associated with the intrinsic timescale estimated using the original ACF-sum method. The regional age effects from the short-lag component were moderately associated with age effects from the original method. **(b)** In contrast, the short-lag component was highly associated with lag-1 temporal autocorrelation (lag-1 TA), both for regional timescale maps and regional age effects. **(c)** The long-lag component and its age effects were strongly associated with the original findings estimated using the ACF-sum method. **(d)** In contrast, the long-lag component was not significantly associated with lag-1 TA in regional timescale maps and was only weakly associated with lag-1 TA regional age effects. Each data point in the scatter plot represents a brain region from the Schaefer 400 atlas. The data points are colored based on their rankings on the S-A axis. *r*_s_ denotes Spearman’s rank correlation coefficient. Significance was assessed using 10,000 spin tests. Linear regression lines are added for visualization purposes only.

*3. The conclusion that "the distribution of INT is hierarchically organized in youth" seems overstated. In the HBN dataset, the association between INT and the sensorimotor-association axis was not significant, which raises concerns about how robust this conclusion is across datasets.*

We agree with the Reviewer that the statement that “the distribution of intrinsic timescale is hierarchically organized in youth” may be too broad, given that the continuous association between intrinsic timescale and the S-A axis was statistically significant in HCPD but not in HBN. We have therefore revised the wording throughout the manuscript to more accurately reflect these findings, focusing on the evidence that timescales are generally longer in the association cortex than in sensorimotor cortex based on the categorical comparisons. The revised manuscript reflects the following changes:

“Results” section, “Intrinsic timescale is hierarchically organized in youth” subsection:

New title of the subsection in the revised manuscript:

Intrinsic timescale is longer in association cortex compared to sensorimotor cortex in youth

And in the same subsection:

Original submission:

Collectively, these findings indicate that the intrinsic timescale is hierarchically organized in youth, with longer timescales observed in association cortex compared to sensorimotor cortex.

Revised manuscript:

Collectively, these findings indicate that the intrinsic timescale tends to be longer in the association cortex compared to the sensorimotor cortex in youth.

New title of Figure 2 in the revised manuscript:

Intrinsic timescale is longer in association cortex compared to sensorimotor cortex in youth

“Discussion” section, paragraph #1:

Original manuscript:

We found that intrinsic timescales are organized along a hierarchical cortical axis (S–A), with longer timescales in association cortex and shorter timescales in sensorimotor cortex.

Revised manuscript:

We found that intrinsic timescales are generally longer in the association cortex and shorter in the sensorimotor cortex in youth.

*4. It is unclear whether site effects were removed or modeled before the GAM analyses. Given the multi-site nature of HCP-D and HBN datasets, this should be explicitly addressed.*

We thank the Reviewer for raising this important point. We agree that site effects should be explicitly addressed. To evaluate whether our findings were driven by data acquisition site differences in HCPD and HBN, we repeated the analyses after harmonizing intrinsic timescale across sites using CovBat-GAM.

Briefly, data acquisition sites were treated as batches, and harmonization was performed while preserving age and sex effects. We then repeated the main age analyses using the harmonized timescale data. Findings were almost perfectly correlated before and after harmonization for both HCPD and HBN datasets (**Figure S7**). These results suggest that the main findings were not driven by site effects.

We have added a new Methods section describing the site harmonization analysis and a supplementary figure showing that the results were highly consistent before and after harmonization. We have also included this information in the Results section.

“Results” section, “Sensitivity analysis” subsection:

Additionally, to ensure that findings were independent from data acquisition sites for HCPD and HBN, we repeated the analyses after harmonizing intrinsic timescale across sites. Findings were almost perfectly correlated before and after harmonization for both HCPD and HBN datasets (**Figure S7**).

“Methods” section:

**Data acquisition site harmonization**

To evaluate whether the findings were influenced by acquisition site, we harmonized intrinsic timescale estimates across data acquisition sites using CovBat-GAM (Johnson et al., 2007; Fortin et al., 2017; Fortin et al., 2018; Pomponio et al., 2020; Chen et al., 2022). Harmonization was performed in R 4.2.2 using the covfam function from the ComBatFamily package. This approach extends ComBat/CovBat harmonization to account for both mean and covariance differences across batches. In this analysis, the study site was treated as the batch variable. Age was included as a smooth term to preserve linear and nonlinear age-related effects, and sex was included as a linear covariate. To assess whether harmonization influenced our findings, we repeated the main analyses before and after site harmonization and compared the resulting whole-brain age associations, regional timescale maps, and regional age-effect maps.

**Figure S7. Findings were robust to site harmonization |** To evaluate whether the findings were influenced by acquisition sites, we harmonized intrinsic timescale estimates across study sites using CovBat-GAM. Age effects (i.e., partial *R*^2^) were highly consistent before and after site harmonization. Each data point in the scatter plots represents a brain region from the Schaefer 400 atlas. The data points are colored based on their rankings on the S-A axis. *r*_s_ denotes Spearman’s rank correlation coefficient. Significance was assessed using 10,000 spin tests. Linear regression lines are added for visualization purposes only.

*5. The adult analysis could be better integrated into a unified developmental model. If the goal is to determine whether developmental effects persist into adulthood, the HCP-D sample alone may already be sufficient, given that its 18-22 year age range captures early adulthood. One could then identify the age window(s) in which INT exhibits significant age‑related change—for example, by using the first derivative of the fitted age trajectory(Sydnor et al. 2023). Alternatively, the HCP‑D and HCP datasets could be combined within a single GAM framework. This approach would likely be more informative than analyzing adults separately. The adult‑only analysis may be more appropriate for the Supplementary Materials.*

We thank the Reviewer for this helpful suggestion. We agree that the adult analysis could be better integrated with the developmental analyses and that the HCPD/HBN age range already provides information about early adulthood. Our goal in including HCPYA was not to infer a continuous developmental trajectory from childhood through adulthood across datasets, but rather to assess the specificity of the age-related patterns observed in youth. In other words, HCPYA was included as an independent young-adult comparison sample to test whether similar age-related associations with intrinsic timescale were still evident during young adulthood.

Following the Reviewer’s suggestion, we added a derivative-based analysis to more directly characterize the timing of age-related differences within the developmental cohorts themselves. Specifically, we estimated the first derivative of each region’s GAM smooth function for age, which provides the age-specific slope of the timescale-age association (Sydnor et al., 2023). This allowed us to identify the age at which each region showed the largest age-related difference in intrinsic timescale and to estimate when the whole-cortex pattern of age-related timescale differences was maximally aligned with the S-A axis. These analyses are now shown in the revised **Figure S2**. We found that the alignment between age-related timescale differences and the S-A axis peaked in early-to-mid adolescence in both developmental datasets, with maximum alignment at 14.6 years in HCPD and 12.8 years in HBN. Thus, the revised analyses suggest that the hierarchical pattern of age-related timescale differences is most prominent in the adolescent period covered by HCPD and HBN (rather than HCPYA).

We opted not to combine HCPD and HCPYA within a single GAM framework. These datasets have both different acquisition parameters (e.g., TR) and a different age range. Under these conditions, a single combined model would make it difficult to separate age-related effects from technical variance across datasets. Similarly, harmonization across datasets with limited age overlap could risk removing or distorting the empirical age-related differences we aim to characterize. We therefore believe the more conservative approach is to model developmental datasets directly and use HCPYA only as an independent young-adult specificity analysis.

Following the Reviewer’s suggestion, we have revised the manuscript to better clarify the role of the HCPYA analysis. We now present the adult analysis more cautiously as a secondary analysis testing whether the age-related associations observed in youth are also evident during young adulthood, rather than as evidence for a continuous lifespan trajectory.

“Results” section, “Timescale remains relatively stable in adulthood” subsection:

Overall, these results emphasize the specificity of the developmental findings in HCPD and HBN to the developmental period of 8 to 22 years old, suggesting that fMRI intrinsic timescale develops along the S–A axis in youth and stabilizes in adulthood. Given the differences in acquisition parameters and cohort characteristics, the young adult dataset was used to assess the specificity of the developmental findings to the developmental cohorts and the stability of age-related effects within young adulthood, rather than as a direct continuation of the developmental age trajectories.

“Discussion” section, paragraph #1:

Moreover, findings in a young adult sample demonstrated that age-related differences in the intrinsic timescale are specific to the developmental cohorts and that the intrinsic timescale appears to remain stable in young adulthood.

We also added the derivative-based timing analysis described above to more directly characterize when age-related differences in intrinsic timescale are most pronounced within the developmental cohorts. These changes are reflected in the revised manuscript.

“Results” section, “Findings replicate in an independent developmental cohort” subsection:

For regions with at least one significant derivative, we summarized the timing of maximal development as the age at which the regional model derivative reached its maximum absolute value (**Figure S2a**). These estimates should be interpreted cautiously because age of maximal development is only defined for regions with significant derivative-based change; regions with weak, stable, or uncertain age trajectories do not have a well-defined peak-change age. To complement this analysis, we also examined the age at which whole-cortex rates of timescale differences were maximally aligned with the S-A axis, which peaked in early-to-mid adolescence in both datasets (**Figure S2b**; 14.6 years in HCPD and 12.8 years in HBN).

“Methods” section:

**Age of maximum alignment between age-related timescale differences and the S-A axis**

To assess when age-related differences in intrinsic timescale were most strongly aligned with the cortical hierarchy, we estimated age-specific rates of timescale differences from the first derivative of each region’s GAM smooth function for age (Sydnor et al., 2023). These derivatives represent the estimated rate of timescale differences per one-year age interval. For each age from 8 to 22 years, we quantified the spatial association between regional derivative values and regional S-A axis ranks using Spearman’s rank correlation. To account for uncertainty in derivative estimates, we sampled 10,000 draws from the posterior distribution of the derivative for each regional GAM smooth and computed the age-specific correlation between derivative values and S-A axis ranks for each draw. The median correlation across draws was used to summarize the age-specific alignment between timescale differences and the S-A axis, and the 95% credible interval was calculated from the distribution of sampled correlations. We then identified the age of maximum alignment as the age at which the derivative-to-S-A axis correlation was maximal for each posterior draw. The median and 95% credible interval of these ages were used to summarize the timing of maximum alignment between age-related timescale differences and the S-A axis.

**Figure S2. Timing of age-related differences in intrinsic timescale across two developmental cohorts |** **(a)** Derivatives of the intrinsic timescale regional developmental patterns (**Figure 3d** and **Figure 4d**) were calculated as finite differences between consecutive time points to estimate the age at which timescale development demonstrated maximal change. Specifically, the age of peak change was estimated as the age where the derivative was greatest in absolute magnitude. **(b)** To estimate the age at which age-related timescale differences were maximally aligned with the S-A axis, we calculated the first derivative of each region’s GAM smooth function for age. These derivatives represent the estimated rate of timescale differences per one-year age interval. At each age, we quantified the spatial correlation between regional derivative values and regional S-A axis ranks. To account for uncertainty in derivative estimates, we sampled 10,000 draws from the posterior derivative distribution and calculated the derivative-to-SA axis correlation for each draw. The black line shows the median correlation across draws, and the gray band shows the 95% credible interval. The pink band shows the 95% credible interval for the age of maximum alignment. Maximal alignment occurred at 14.6 years in HCPD and 12.8 years in HBN.

*6. The reported age-related increase in INT appears inconsistent with previous studies reporting developmental decreases. Although the authors attribute this discrepancy to differences in imaging modality, preprocessing, and analytic approaches, these explanations also raise questions regarding the cross-study robustness and interpretability of INT measures. The authors should discuss this issue more clearly.*

We thank the Reviewer for this comment. We agree that the apparent discrepancy between our age-related increases in fMRI intrinsic timescale and prior reports of developmental decreases should be discussed more clearly, especially because such differences raise broader questions about the interpretation of intrinsic timescale across modalities and analytic choices.

In the revised manuscript, we have expanded the Discussion to more explicitly address this issue. We now emphasize that intrinsic timescale measures are not interchangeable across modalities, preprocessing pipelines, and metric definitions. Specifically, our primary ACF-sum measure was estimated from band-pass filtered fMRI data (0.01-0.08Hz). Because temporal filtering influences the shape of the autocorrelation function, restricting the signal to slower BOLD fluctuations can produce a more slowly decaying ACF, making the ACF-sum measure particularly sensitive to longer-lag positive autocorrelation. In contrast, prior electrophysiological studies (e.g., EEG, ECoG) capture faster neural dynamics over broader frequency ranges, where developmental decreases in timescale may reflect maturation-related changes in fast local electrophysiological dynamics. Thus, the association-cortex increases observed in our study could reflect increasing persistence of slow network-level BOLD dynamics across childhood and adolescence, rather than necessarily contradicting evidence for faster local neural dynamics with maturation.

We have also added new supplementary analyses to clarify the interpretability of different intrinsic timescale metrics within our own data. First, we repeated our HCPD analysis using lag-1 temporal autocorrelation, following Bero et al. (2026), and found partially overlapping but non-identical developmental patterns compared with our original ACF-sum measure. Second, we decomposed the ACF-sum metric into short- and long-lag components, showing that lag-1 TA primarily reflects short-lag temporal persistence, whereas ACF-sum timescale is more sensitive to sustained positive autocorrelation across longer lags. These findings support the view that different intrinsic timescale definitions can capture related but distinct properties of the signal.

We have now revised the Discussion to frame this interpretation more cautiously. Specifically, we state that methodological differences in modality, preprocessing, and metric definition may contribute to discrepancies across studies, but that these possibilities remain speculative and should be tested directly in future multimodal studies using matched timescale definitions and harmonized preprocessing pipelines. The revised manuscript reflects these additions:

“Discussion” section, new paragraph #4:

To further investigate these differences, we repeated our developmental analyses using lag-1 TA in the same HCPD sample and preprocessing pipeline used for our primary ACF-sum analyses. This comparison showed that lag-1 TA and ACF-sum timescale had distinct regional mean maps but demonstrated partially overlapping regional age effects. Decomposing the ACF-sum timescale into short- and long-lag components further suggested that lag-1 TA and ACF-sum timescale emphasize partially distinct features of the ACF: lag-1 TA primarily reflects immediate short-lag decorrelation, whereas ACF-sum timescale is more sensitive to the broader shape of the ACF, including sustained positive autocorrelation across longer lags. Thus, while methodological differences in preprocessing, sample selection, and modeling may contribute to discrepancies across studies, our results indicate that some differences may also arise because commonly used timescale metrics emphasize different characteristics of the same fMRI signal.

“Discussion” section, paragraph #6:

Furthermore, prior studies using imaging modalities with higher temporal resolution, such as EEG and ECoG, have reported overall decreases in intrinsic timescale during development (McKeon et al., 2025; Miles et al., 2026). Although similar measures of timescale were used across these studies and our study in fMRI, the overall age-related trends in timescale varied across modalities. These differences may reflect the fact that different imaging modalities probe distinct aspects of neural dynamics, as they are sensitive to different frequency ranges and may preferentially capture activity arising from different laminar sources (Scheeringa et al., 2016; Sadaghiani & Wirsich, 2020). In this context, developmental decreases in electrophysiological timescale and increases in fMRI-based ACF-sum timescale may not necessarily reflect opposing developmental patterns. Instead, electrophysiological studies may be more sensitive to faster local neural dynamics, whereas the present fMRI-based measure reflects the persistence of slow BOLD fluctuations within the 0.01-0.08 Hz range. Because temporal filtering influences the shape of the autocorrelation function (Shumway & Stoffer, 2017), restricting the signal to slower BOLD fluctuations can produce a more slowly decaying ACF. Consistent with this interpretation, our short- and long-lag decomposition of the fMRI autocorrelation function suggests that the ACF-sum measure is particularly sensitive to longer-lag positive autocorrelation, indicating that the association-cortex increases observed here should not be interpreted as a global slowing of neural activity across all temporal scales. Finally, discrepancies across studies, even within the same modality, may also arise from differences in data acquisition, preprocessing pipelines, and analytic approaches. Such methodological variation can influence estimates of resting-state dynamics (e.g., time series properties, cross-correlations, and intrinsic timescale; Li et al., 2024) and should be considered when interpreting findings.

*7. It would be helpful to report model statistics, such as R² and p-values, directly on the age-trajectory figures.*

Thank you for this suggestion. We have now added model statistics (i.e., partial *R*² and *p*-values) to the figures.

## Added references:

Chen, A. A., Beer, J. C., Tustison, N. J., Cook, P. A., Shinohara, R. T., & Shou, H. (2022). Mitigating site effects in covariance for machine learning in neuroimaging data. *Human Brain Mapping*, *43*(4), 1179–1195. https://doi.org/10.1002/hbm.25688

Fortin, J.-P., Cullen, N., Sheline, Y. I., Taylor, W. D., Aselcioglu, I., Cook, P. A., Adams, P., Cooper, C., Fava, M., McGrath, P. J., McInnis, M., Phillips, M. L., Trivedi, M. H., Weissman, M. M., & Shinohara, R. T. (2018). Harmonization of cortical thickness measurements across scanners and sites. *NeuroImage*, *167*, 104–120. https://doi.org/10.1016/j.neuroimage.2017.11.024

Fortin, J.-P., Parker, D., Tunç, B., Watanabe, T., Elliott, M. A., Ruparel, K., Roalf, D. R., Satterthwaite, T. D., Gur, R. C., Gur, R. E., Schultz, R. T., Verma, R., & Shinohara, R. T. (2017). Harmonization of multi-site diffusion tensor imaging data. *NeuroImage*, *161*, 149–170. https://doi.org/10.1016/j.neuroimage.2017.08.047

Johnson, W. E., Li, C., & Rabinovic, A. (2007). Adjusting batch effects in microarray expression data using empirical Bayes methods. *Biostatistics*, *8*(1), 118–127. https://doi.org/10.1093/biostatistics/kxj037

Shinn, M., Hu, A., Turner, L., Noble, S., Preller, K. H., Ji, J. L., Moujaes, F., Achard, S., Scheinost, D., Constable, R. T., Krystal, J. H., Vollenweider, F. X., Lee, D., Anticevic, A., Bullmore, E. T., & Murray, J. D. (2023). Functional brain networks reflect spatial and temporal autocorrelation. *Nature Neuroscience*, *26*(5), 867–878. https://doi.org/10.1038/s41593-023-01299-3

Shumway, R. H., & Stoffer, D. S. (2017). *Time series analysis and its applications*. 4th edition: Springer International Publishing. https://doi.org/10.1007/978-3-319-52452-8
