# Supplementary material for: Intrinsic timescale develops hierarchically from sensorimotor to association cortex in youth and stabilizes in adulthood
Source: PLoS Biol. 2026 Sep 29;24(9):e3004037. doi: 10.1371/journal.pbio.3004037 (PMC13638041; doi:10.1371/journal.pbio.3004037)
Supplement: Peer review history

# Editor report: Decision Letter 0

**DOI:** 10.1371/journal.pbio.3004037.r001

**Authors:** Christian Schnell, PhD

15 Apr 2026

Dear Dr Shafiei,

Thank you for submitting your manuscript entitled "Mapping developmental patterns of intrinsic timescale " for consideration as a Research Article by PLOS Biology.

Your manuscript has now been evaluated by the PLOS Biology editorial staff as well as by an academic editor with relevant expertise and I am writing to let you know that we would like to send your submission out for external peer review.

However, before we can send your manuscript to reviewers, we need you to complete your submission by providing the metadata that is required for full assessment. To this end, please login to Editorial Manager where you will find the paper in the 'Submissions Needing Revisions' folder on your homepage. Please click 'Revise Submission' from the Action Links and complete all additional questions in the submission questionnaire.

Once your full submission is complete, your paper will undergo a series of checks in preparation for peer review. After your manuscript has passed the checks it will be sent out for review. To provide the metadata for your submission, please Login to Editorial Manager (https://www.editorialmanager.com/pbiology) within two working days, i.e. by Apr 17 2026 11:59PM.

If your manuscript has been previously peer-reviewed at another journal, PLOS Biology is willing to work with those reviews in order to avoid re-starting the process. Submission of the previous reviews is entirely optional and our ability to use them effectively will depend on the willingness of the previous journal to confirm the content of the reports and share the reviewer identities. Please note that we reserve the right to invite additional reviewers if we consider that additional/independent reviewers are needed, although we aim to avoid this as far as possible. In our experience, working with previous reviews does save time.

If you would like us to consider previous reviewer reports, please edit your cover letter to let us know and include the name of the journal where the work was previously considered and the manuscript ID it was given. In addition, please upload a response to the reviews as a 'Prior Peer Review' file type, which should include the reports in full and a point-by-point reply detailing how you have or plan to address the reviewers' concerns.

During the process of completing your manuscript submission, you will be invited to opt-in to posting your pre-review manuscript as a bioRxiv preprint. Visit http://journals.plos.org/plosbiology/s/preprints for full details. If you consent to posting your current manuscript as a preprint, please upload a single Preprint PDF.

Feel free to email us at plosbiology@plos.org if you have any queries relating to your submission.

Kind regards,

Christian

Christian Schnell, PhD

Senior Editor

PLOS Biology

cschnell@plos.org

# Peer review: Decision Letter 1

**DOI:** 10.1371/journal.pbio.3004037.r002

**Authors:** Christian Schnell, PhD

21 May 2026

Dear Dr Shafiei,

Thank you for your patience while your manuscript "Mapping developmental patterns of intrinsic timescale " was peer-reviewed at PLOS Biology. It has now been evaluated by the PLOS Biology editors, an Academic Editor with relevant expertise, and by several independent reviewers.

In light of the reviews, which you will find at the end of this email, we would like to invite you to revise the work to thoroughly address the reviewers' reports.

As you will see below, the reviewers write that study is overall well executed and provides potentially important insights. However, they also raise a number of concerns that need to be addressed. For Reviewer 1 and 2, the most important comments are on the discrepancies with prior work on intrinsic timescale development. There are also concerns about the use of a different metric by Reviewer 3, and about the framing/terminology around “development changes” in this cross-sectional study.

Given the extent of revision needed, we cannot make a decision about publication until we have seen the revised manuscript and your response to the reviewers' comments. Your revised manuscript is likely to be sent for further evaluation by all or a subset of the reviewers.

In addition to these revisions, you will need to complete some formatting changes, which you will receive in a follow up email. A member of our team will be in touch with a set of requests shortly.

We expect to receive your revised manuscript within 3 months. Please email us (plosbiology@plos.org) if you have any questions or concerns, or would like to request an extension.

At this stage, your manuscript remains formally under active consideration at our journal; please notify us by email if you do not intend to submit a revision so that we may withdraw it.

**IMPORTANT - SUBMITTING YOUR REVISION**

Your revisions should address the specific points made by each reviewer. Please submit the following files along with your revised manuscript:

1. A 'Response to Reviewers' file - this should detail your responses to the editorial requests, present a point-by-point response to all of the reviewers' comments, and indicate the changes made to the manuscript.

*NOTE: In your point-by-point response to the reviewers, please provide the full context of each review. Do not selectively quote paragraphs or sentences to reply to. The entire set of reviewer comments should be present in full and each specific point should be responded to individually, point by point.

You should also cite any additional relevant literature that has been published since the original submission and mention any additional citations in your response.

2. In addition to a clean copy of the manuscript, please also upload a 'track-changes' version of your manuscript that specifies the edits made. This should be uploaded as a "Revised Article with Changes Highlighted" file type.

*Re-submission Checklist*

When you are ready to resubmit your revised manuscript, please refer to this re-submission checklist: https://plos.io/Biology_Checklist

To submit a revised version of your manuscript, please go to https://www.editorialmanager.com/pbiology/ and log in as an Author. Click the link labelled 'Submissions Needing Revision' where you will find your submission record.

Please make sure to read the following important policies and guidelines while preparing your revision:

*Published Peer Review*

Please note while forming your response, if your article is accepted, you may have the opportunity to make the peer review history publicly available. The record will include editor decision letters (with reviews) and your responses to reviewer comments. If eligible, we will contact you to opt in or out. Please see here for more details:

https://blogs.plos.org/plos/2019/05/plos-journals-now-open-for-published-peer-review/

*PLOS Data Policy*

Please note that as a condition of publication PLOS' data policy (http://journals.plos.org/plosbiology/s/data-availability) requires that you make available all data used to draw the conclusions arrived at in your manuscript. If you have not already done so, you must include any data used in your manuscript either in appropriate repositories, within the body of the manuscript, or as supporting information (N.B. this includes any numerical values that were used to generate graphs, histograms etc.). For an example see here: http://www.plosbiology.org/article/info%3Adoi%2F10.1371%2Fjournal.pbio.1001908#s5

*Blot and Gel Data Policy*

We require the original, uncropped and minimally adjusted images supporting all blot and gel results reported in an article's figures or Supporting Information files. We will require these files before a manuscript can be accepted so please prepare them now, if you have not already uploaded them. Please carefully read our guidelines for how to prepare and upload this data: https://journals.plos.org/plosbiology/s/figures#loc-blot-and-gel-reporting-requirements

*Protocols deposition*

To enhance the reproducibility of your results, we recommend that if applicable you deposit your laboratory protocols in protocols.io, where a protocol can be assigned its own identifier (DOI) such that it can be cited independently in the future. Additionally, PLOS ONE offers an option for publishing peer-reviewed Lab Protocol articles, which describe protocols hosted on protocols.io. Read more information on sharing protocols at https://plos.org/protocols?utm_medium=editorial-email&utm_source=authorletters&utm_campaign=protocols

Thank you again for your submission to our journal. We hope that our editorial process has been constructive thus far, and we welcome your feedback at any time. Please don't hesitate to contact us if you have any questions or comments.

Sincerely,

Christian

Christian Schnell, PhD

Senior Editor

PLOS Biology

cschnell@plos.org

------------------------------------

REVIEWS:

Reviewer #1 (Jesse T. Miles signed his report): Summary of the research and overall impression

This manuscript reports a developmental shift in intrinsic neural timescales organized around the sensorimotor-to-association neural hierarchy, otherwise known as the SA axis. Specifically, the authors report an increase in neural timescales throughout childhood and adolescence which demonstrates stronger age-dependence in association cortex, effect-size increases that recapitulate the SA axis, and greater timescale stability in sensorimotor regions. Based on these results the authors claim that sensorimotor region intrinsic neural timescales are relatively stable throughout childhood and adolescence, while timescales increase in association regions.

A strength of the manuscript is that the authors reproduce their findings using two large, independent developmental fMRI datasets, and show that the age-dependence does not continue into young adulthood in a third dataset. The authors also diligently varied analytic workflows, and in doing so demonstrated that their conclusions were robust to a variety of possible demographic and analytic confounds. In addition, the manuscript is well-organized, well-researched, and impressively up-to-date.

However, more discussion of the fact that the conclusions seem to differ from prior work on intrinsic timescale development is warranted. The authors point out certain methodological and demographic differences between their work and others, but the studies cited used a wide variety of neural recording modalities, preprocessing techniques, analytic pipelines, and age ranges to come to broadly similar conclusions (i.e., timescales generally decrease with age, but may do so heterogeneously across the cortex in ways that align with SA axis maturation). Because of the broad interest of the work, its efforts toward methodological rigor, and its potential novelty, I recommend the manuscript be accepted. However, explaining and recontextualizing its findings may require major revision.

---

Specific areas for improvement

1. The Bero et al., 2026 study that the authors cite did not find relationships between childhood timescales and the S-A axis (figure 13), despite finding that some frontal regions may increase their temporal timescales throughout childhood (figure 8). While many frontal regions rank high on the SA axis, other high-ranking regions are not in the frontal cortex. Plus, Bero et al. report generally faster timescales in adults than children (figure 2), even in many networks that contain frontal regions. The authors point out that Bero et al. used a different timescale measure, which could account for some differences, but the analytic methods seem similar enough that I'm surprised the conclusions differ. Can the authors comment on whether they, too, see their results as primarily applicable to frontal regions instead of association regions, broadly? Aside from methodological differences, are there other reasons that could explain the differences between their findings and Bero et al.'s?

>Suggested analyses to strengthen the link to Bero et al., 2026:

Running a supplemental analysis using the lag-1 autocorrelation. The authors already compare their method to fitting an exponential decay and to calculating the first zero-crossing of the ACF window, so they seem to already be set up to make the lag-1 comparison. I also think it's important to run at least one dataset through the entire analysis, not just compare the timescales output by the different methods. Essentially, reproduce figure 3 with the lag-1 timescales to establish whether Bero et al.'s method would lead to a different conclusion. If it doesn't then clearly there is more driving the differences than the timescale calculation.

2. The authors mention that Truzzi and Cusack, 2023, found differences in the timescale hierarchy between infants and adults, and found that adult timescales were generally much shorter. In this manuscript, the authors report similar timescale hierarchies between adults and children/adolescents, while Truzzi and Cusack 2023 suggest that timescales decrease globally. The authors seem to suggest that this is because the SA axis becomes more developed between the neonatal period and middle childhood, giving it "adult-like hierarchical ordering". Similar results to Truzzi and Cusack, 2023 were found in McKeon et al., 2025, who used a similar age range in their EEG and sEEG studies, and Miles et al., 2026, who found association region timescale decreases throughout adolescence. Why, then, do the authors think that they see timescale increases in association regions?

3. Comparing the authors own results in the manuscript, it appears that the independent adult sample had significantly shorter timescales (ranging from ~3.7-4.4 s) than either of the developmental samples (timescales ranging from ~4.6 - 5.5 s). I understand that the authors are more focused on the lack of age-dependent changes in the adult sample. Still, the fact that timescales were generally found to be lower - both globally and regionally - in the adult sample needs to be reconciled with the authors' claims that association region timescales increase throughout adolescence and remain stable in sensorimotor areas.

>Suggested analyses to test whether sensorimotor timescales continue diverging from association regions during middle childhood/adolescence:

A) Testing whether timescale differences between regions near opposing poles of the SA axis become increasingly larger with age than differences between regions at similarly situated points of the SA axis. In other words: are unimodal region timescales more like each other than they are to transmodal regions? Does the size of the difference between the unimodal-to-unimodal comparisons and the unimodal-to-transmodal comparisons depend on age? The null hypothesis here would be that the within and across hierarchy timescale comparisons do not change throughout adolescence. Age-dependent increases, however, would align with a model of increased SA timescale differentiation. This analysis would be especially impactful if done using within-subject SA axis comparisons, though would not necessarily help to establish whether any differences were due to sensorimotor timescale decreases, association timescale increases, or differences in relative rates of change.

B) Assessing correlations between age-of-peak-change and SA axis. The hypothesis that sensorimotor region timescales develop early and remain stable throughout development predicts lower ages-of-peak-change and smaller derivatives relative to association regions. This could be an addition to supplemental figure 1, which already calculates age-to-peak change, and would supplement the prior suggested analysis.

---

While the specific analyses suggested should be thought of as recommendations, not requirements, further effort to clarify the relationship between the authors' results and the current state-of-the-field will be essential to support their conclusions.

I look forward to seeing how the manuscript develops in revision and commend the authors for a thoughtful and timely submission.

Reviewer #2 (Hoyt Patrick Taylor IV signed his report): The authors carry out an analysis of intrinsic timescale in fMRI using the HCPD, HBN, and HCPYA cohorts. They find that development of intrinsic timescale as measured using ACF plays out along the S-A axis during youth, driven primarily by increasing timescales in association cortex.

The replication analysis and the inclusion of the HCPYA cohort are clear strengths. The authors candidly engage with a recent paper (Bero et al. 2026) which also analyzed timescale in the HCPD cohort. The findings of Bero et al. 2026 are at odds with the findings of this work, which I believe requires more rigorous treatment from the authors.

This manuscript reports stable intrinsic timescale in sensorimotor cortex and increases in association cortex, while Bero et al. reports decreases in sensorimotor and increases in prefrontal cortex. Authors propose that this discrepancy is accounted for by the fact that the full ACF captures different dynamics than lag-1 TA, however I think the manuscript would benefit from further analyzing this hypothesis.

I would encourage the authors to compute lag-1 TA on the same preprocessed HCPD data and demonstrate the relationship to their ACF-sum metric, verifying the Bero et al finding of decreasing lag-1 TA in sensorimotor cortex. If the ACF sum could be decomposed into short-lag and longer-lag contributions, this could potentially reveal the mechanism for the discrepancy between these metrics.

If the authors opt not to carry out the above analysis, the discussion should at least be expanded to acknowledge that the disagreement is not mechanistically understood and to outline what would be necessary to probe the mechanism empirically.

Outside of this concern, I am convinced by the conclusions of the paper.

Reviewer #3: The manuscript addresses an interesting topic by characterizing the spatial distribution and developmental trajectories of intrinsic neural timescales (INT). However, I have several concerns regarding the framing, methodological justification, and strength of the conclusions.

1. The rationale that previous reports of lifespan changes in neural dynamics (Bero et al., 2026) motivate the present analysis also requires further clarification, particularly the manuscript does not offer a clear conceptual and methodological advance beyond prior work.

2. The rationale for using a full-shape autocorrelation-based INT measure should be clarified. Alternative definitions, including ACF decay time to a fixed threshold(0.5 or 1/e) (Raut et al. 2020; Gao et al. 2020), lag-1 temporal autocorrelation (Bero et al. 2026) and spectral parametrization(Gao et al. 2020), are widely used. Since the authors acknowledge in the Discussion that different INT definitions may capture partially overlapping but distinct aspects of neural dynamics, it would be valuable to examine the extent to which the main spatial and developmental findings converge or diverge across alternative INT metrics within the same analytic framework.

3. The conclusion that "the distribution of INT is hierarchically organized in youth" seems overstated. In the HBN dataset, the association between INT and the sensorimotor-association axis was not significant, which raises concerns about how robust this conclusion is across datasets.

4. It is unclear whether site effects were removed or modeled before the GAM analyses. Given the multi-site nature of HCP-D and HBN datasets, this should be explicitly addressed.

5. The adult analysis could be better integrated into a unified developmental model. If the goal is to determine whether developmental effects persist into adulthood, the HCP-D sample alone may already be sufficient, given that its 18-22 year age range captures early adulthood. One could then identify the age window(s) in which INT exhibits significant age‑related change—for example, by using the first derivative of the fitted age trajectory(Sydnor et al. 2023). Alternatively, the HCP‑D and HCP datasets could be combined within a single GAM framework. This approach would likely be more informative than analyzing adults separately. The adult‑only analysis may be more appropriate for the Supplementary Materials.

6. The reported age-related increase in INT appears inconsistent with previous studies reporting developmental decreases. Although the authors attribute this discrepancy to differences in imaging modality, preprocessing, and analytic approaches, these explanations also raise questions regarding the cross-study robustness and interpretability of INT measures. The authors should discuss this issue more clearly.

7. It would be helpful to report model statistics, such as R² and p-values, directly on the age-trajectory figures.

# Author comment: Author response to Decision Letter 2

**DOI:** 10.1371/journal.pbio.3004037.r003

14 Aug 2026

Attachment Submitted filename: Shafiei_timescale_plosbiology_response_letter.docx

# Peer review: Decision Letter 2

**DOI:** 10.1371/journal.pbio.3004037.r004

**Authors:** Christian Schnell, PhD

7 Sep 2026

Dear Dr Shafiei,

Thank you for your patience while we considered your revised manuscript "Mapping developmental patterns of intrinsic timescale " for publication as a Research Article at PLOS Biology. This revised version of your manuscript has been evaluated by the PLOS Biology editors, the Academic Editor and one of the original reviewers.

Based on the reviews and on our Academic Editor's assessment of your revision, we are likely to accept this manuscript for publication, provided you satisfactorily address the following data and other policy-related requests:

* We would like to suggest a different title to improve its accessibility for our broad audience:

"Intrinsic timescale develops hierarchically from sensorimotor to association cortex in youth and stabilizes in adulthood"

* Please add the links to the funding agencies in the Financial Disclosure statement in the manuscript details.

* DATA POLICY:

You may be aware of the PLOS Data Policy, which requires that all data be made available without restriction: http://journals.plos.org/plosbiology/s/data-availability. For more information, please also see this editorial: http://dx.doi.org/10.1371/journal.pbio.1001797

Note that we do not require all raw data. Rather, we ask that all individual quantitative observations that underlie the data summarized in the figures and results of your paper be made available in one of the following forms:

1) Supplementary files (e.g., excel). Please ensure that all data files are uploaded as 'Supporting Information' and are invariably referred to (in the manuscript, figure legends, and the Description field when uploading your files) using the following format verbatim: S1 Data, S2 Data, etc. Multiple panels of a single or even several figures can be included as multiple sheets in one excel file that is saved using exactly the following convention: S1_Data.xlsx (using an underscore).

2) Deposition in a publicly available repository. Please also provide the accession code or a reviewer link so that we may view your data before publication.

Regardless of the method selected, please ensure that you provide the individual numerical values that underlie the summary data displayed in the following figure panels as they are essential for readers to assess your analysis and to reproduce it: 2B and 6A.

NOTE: the numerical data provided should include all replicates AND the way in which the plotted mean and errors were derived (it should not present only the mean/average values).

Please also ensure that figure legends in your manuscript include information on where the underlying data can be found, and ensure your supplemental data file/s has a legend.

Please ensure that your Data Statement in the submission system accurately describes where your data can be found.

* CODE POLICY

Per journal policy, if you have generated any custom code during the course of this investigation, please make it available without restrictions. Please ensure that the code is sufficiently well documented and reusable, and that your Data Statement in the Editorial Manager submission system accurately describes where your code can be found. More information on our Code Policy, what and how to share can be found here: https://journals.plos.org/plosbiology/s/code-availability

Please note that we cannot accept sole deposition of code in GitHub, as this could be changed after publication. However, you can archive this version of your publicly available GitHub code to Zenodo. Once you do this, it will generate a DOI number, which you will need to provide in the Data Accessibility Statement (you are welcome to also provide the GitHub access information). See the process for doing this here: https://docs.github.com/en/repositories/archiving-a-github-repository/referencing-and-citing-content

As you address these items, please take this last chance to review your reference list to ensure that it is complete and correct. If you have cited papers that have been retracted, please include the rationale for doing so in the manuscript text, or remove these references and replace them with relevant current references. Any changes to the reference list should be mentioned in the cover letter that accompanies your revised manuscript.

In addition to these revisions, you may need to complete some formatting changes, which you will receive in a follow up email. A member of our team will be in touch with a set of requests shortly. If you do not receive a separate email within a few days, please assume that checks have been completed, and no additional changes are required.

We expect to receive your revised manuscript within two weeks.

To submit your revision, please go to https://www.editorialmanager.com/pbiology/ and log in as an Author. Click the link labelled 'Submissions Needing Revision' to find your submission record. Your revised submission must include the following:

- a cover letter that should detail your responses to any editorial requests, if applicable, and whether changes have been made to the reference list

- a Response to Reviewers file that provides a detailed response to the reviewers' comments (if applicable, if not applicable please do not delete your existing 'Response to Reviewers' file.)

- a track-changes file indicating any changes that you have made to the manuscript.

NOTE: If Supporting Information files are included with your article, note that these are not copyedited and will be published as they are submitted. Please ensure that these files are legible and of high quality (at least 300 dpi) in an easily accessible file format. For this reason, please be aware that any references listed in an SI file will not be indexed. For more information, see our Supporting Information guidelines:

https://journals.plos.org/plosbiology/s/supporting-information

*Published Peer Review History*

Please note that you may have the opportunity to make the peer review history publicly available. The record will include editor decision letters (with reviews) and your responses to reviewer comments. If eligible, we will contact you to opt in or out. Please see here for more details:

https://plos.org/published-peer-review-history/

*Press*

Should you, your institution's press office or the journal office choose to press release your paper, please ensure you have opted out of Early Article Posting on the submission form. We ask that you notify us as soon as possible if you or your institution is planning to press release the article.

*Protocols deposition*

To enhance the reproducibility of your results, we recommend that if applicable you deposit your laboratory protocols in protocols.io, where a protocol can be assigned its own identifier (DOI) such that it can be cited independently in the future. Additionally, PLOS ONE offers an option for publishing peer-reviewed Lab Protocol articles, which describe protocols hosted on protocols.io. Read more information on sharing protocols at https://plos.org/protocols?utm_medium=editorial-email&utm_source=authorletters&utm_campaign=protocols

Please do not hesitate to contact me should you have any questions.

Sincerely,

Christian

Christian Schnell, PhD

Senior Editor

cschnell@plos.org

PLOS Biology

------------------------------------------------------------------------

Reviewer remarks:

Reviewer #1 (Jesse T. Miles): The revisions have sufficiently addressed my comments.

# Author comment: Author response to Decision Letter 3

**DOI:** 10.1371/journal.pbio.3004037.r005

17 Sep 2026

Attachment Submitted filename: Shafiei_timescale_plosbiology_response_letter_r1.docx

# Editor report: Decision Letter 3

**DOI:** 10.1371/journal.pbio.3004037.r006

**Authors:** Christian Schnell, PhD

18 Sep 2026

Dear Dr Shafiei,

Thank you for the submission of your revised Research Article "Intrinsic timescale develops hierarchically from sensorimotor to association cortex in youth and stabilizes in adulthood" for publication in PLOS Biology. On behalf of my colleagues and the Academic Editor, Anila D'Mello, I am pleased to say that we can in principle accept your manuscript for publication, provided you address any remaining formatting and reporting issues. These will be detailed in an email you should receive within 2-3 business days from our colleagues in the journal operations team; no action is required from you until then. Please note that we will not be able to formally accept your manuscript and schedule it for publication until you have completed any requested changes.

Please take a minute to log into Editorial Manager at http://www.editorialmanager.com/pbiology/, click the "Update My Information" link at the top of the page, and update your user information to ensure an efficient production process.

PRESS

We frequently collaborate with press offices. If your institution or institutions have a press office, please notify them about your upcoming paper at this point, to enable them to help maximize its impact. If the press office is planning to promote your findings, we would be grateful if they could coordinate with biologypress@plos.org. If you have previously opted in to the early version process, we ask that you notify us immediately of any press plans so that we may opt out on your behalf.

We also ask that you take this opportunity to read our Embargo Policy regarding the discussion, promotion and media coverage of work that is yet to be published by PLOS. As your manuscript is not yet published, it is bound by the conditions of our Embargo Policy. Please be aware that this policy is in place both to ensure that any press coverage of your article is fully substantiated and to provide a direct link between such coverage and the published work. For full details of our Embargo Policy, please visit http://www.plos.org/about/media-inquiries/embargo-policy/.

Thank you again for choosing PLOS Biology for publication and supporting Open Access publishing. We look forward to publishing your study.

Sincerely,

Christian

Christian Schnell, PhD

Senior Editor

PLOS Biology

cschnell@plos.org
